# Supplementary material for: Hierarchical conductive metal-organic framework films enabling efficient interfacial mass transfer
Source: Nat Commun. 2023 Jun 29;14:3850. doi: 10.1038/s41467-023-39630-y (PMC10310809; doi:10.1038/s41467-023-39630-y)
Supplement: Supplementary file 1 — Supplementary Information [file 41467_2023_39630_MOESM1_ESM.pdf]

Supplementary Information

Hierarchical conductive metal-organic framework films enabling efficient interfacial mass transfer

C. Huang et al.

Supplementary Materials:

Supplementary Method

Supplementary Figures and Figure Captions 1 to 51

Supplementary Tables 1 to 5

Supplementary References

Supplementary Method

Materials

All chemicals used were at least of analytical grade. Zinc nitrate hexahydrate ($Zn(NO_3)_2$•$6H_2O$), zinc acetate, Cobalt nitrate hexahydrate ($Co(NO_3)_2\cdot 6H_2O$) and 2-methylimidazole were purchased from Sigma-Aldrich (Germany). 2,3,6,7,10,11-hexahydroxytriphenylene (HHTP) purchased from TCI company. Ultrapure water (18.2 MΩ) produced by a Millipore direct-Q system (Millipore) was used throughout the experiments. Commercial reagents were purchased from Sigma-Aldrich (ACS grade) and used as received unless otherwise noted

Characterisation

Powder X-ray diffraction (PXRD) patterns were obtained on an X-ray diffractometer (Aeris Research Edition, Malvern Panalytical Company) using Cu-Kα radiation (λ = 0.15418 nm) at 40 kV and 15 mA at room temperature. The as-obtained powder samples were measured in reflection geometry. The FT-IR spectra were measured using a Bruker Tensor II IR spectrometer with a universal Zn-Se ATR (attenuated total reflection) accessory in the 500~4000 cm^{-1}. Scanning electron microscopy (SEM) was carried out on a field emission scanning electron microscope (FESEM, Zeiss Gemini 500). Transmission electron microscopy (TEM) measurements were carried out with a Libra120 (Carl Zeiss Microscopy GmbH, Germany). Transmission electron microscopy (TEM) and high-resolution TEM (HRTEM) were performed using a LIBRA 120 MC Cs STEM (Carl Zeiss) operating at an accelerating voltage of 120 kV. The concentration of Zn^{2+} was determined by optical emission spectrometry (OES) with radial observation of inductively coupled plasma (ICP) using SPECTRO Arcos spectrometer. The surface area and pore diameter were determined with a physisorption analyser (model ASAP 2020M; Micromeritics, Norcross, GA, USA) at -196 °C. Before measurements, samples were degassed *in vacuo* at 100 °C for at least 8 h. The Brunner−Emmet−Teller (BET) method was used to calculate the specific surface areas (SBET) using adsorption data at P/P_0 of 0.05–0.30. The pore size distributions (PSDs) were derived from the adsorption branches of the isotherms using the Barrett-Joyner-Halenda (BJH) model. The total pore volume (V_t) was estimated from the adsorbed amount at P/P_0 of 0.995. Thermogravimetric analysis (TGA) was carried out using a Pyris 1 TGA (PerkinElmer, Waltham, MA, USA) with a nitrogen flow of 10 mL min^{-1}.

Computational details for the free energy

The geometric structures of material models with all real frequencies were optimized by using the PBE0 method[1] in conjunction with the 6-31G(d) basis set for C, O, and H atoms and LANL2DZ basis set for Zn atom. In order to simulate the practical structures of materials, the planar structures of Zn-HHTP was maintained during the optimization process by immobilizing the surrounding groups or restricting symmetry optimization. Based on the optimized structures, the Gibbs free energy change (ΔG) for the conversion of ZIF-8 to Zn-HHTP was computed at the same level. All the calculations were performed by using Gaussian 16 software[2], and the structural pictures were plotted by employing Gaussview program[3].

Computational fluid dynamics for gas adsorption in porous media.

Three dimensional numerical simulations of N_2/NH_3 mixture gas are conducted using ANSYS Fluent 19.0 with laminar flow model. NH_3 gas convection/diffusion and adsorption in Zn-HHTP films are modelled with species transport model combined with porous media model and user defined functions (UDFs) to consider the adsorption of NH_3 on porous surfaces. The NH_3 species is initialized at the upper boundary with a unity specified mass fraction. The left boundary is set as a velocity inlet where N_2 gas delivers the NH_3 into the Zn-HHTP films at speed of 100 μm s^{-1}. The right boundary is set as a pressure outlet to allow the N_2/NH_3 mixture flows out of the computation domain. The front and back boundaries are symmetrical ones without any mass flux. All simulations are conducted under room conditions. The computation domain is 2.5 μm×2.5 μm×1.5 μm and the porous media is fixed at the centre of the bottom surface. We generate a hexahedral non-uniform structured mesh (totally about 2.8×10^6 grids). The mesh is refined at the MOF surfaces (Supplementary Figure 32). Mesh convergence is determined by the final adsorption amount along with the maximum adsorption concentration reached in the porous media during flow.

The porous media is considered by adding a momentum source term to the fluid flow governing equations. The viscous loss and inertial loss in the source term are characterized by the viscous resistance factor $1/K_{bulk}$ (m^{-2}, i.e., inverse absolute permeability K_{bulk}) and inertial resistance factor (m^{-1}). In the present study, the inertial loss can be ignored and the permeability K_{bulk} of the porous media is set to 2×10^{-15} m^2.

The adsorption kinetic of NH_3 in porous surfaces is presented by Langmuir kinetic model:

$$\frac{dq}{dt} = k_a C(1-q) - k_d q \tag{S1}$$

where q (mg g^{-1}) is the adsorption concentration, and k_a and k_d are adsorption and desorption rate constants, respectively. Here, q is nondimensionalized by the maximum adsorption capacity q_m, which is proportional to the specific surface area σ. The mass source term in the NH_3 transport governing equation can be expressed as:

$$S_m = -(1-\varepsilon)\rho_p \frac{dq}{dt} \tag{S2}$$

where ρ_p is the solid density of porous media. Two UDFs are built for achieving the dynamic adsorption of NH_3 gas in the porous media. Dynamic adsorption amount dq/dt and mass source S_m are calculated by the equations (S1) and (S2) at every time step, respectively. Dynamic adsorption concentration q is calculated by the time integration at right hand side of equation (S1) using finite difference method at the end of every iteration.

Preparation of *c*-MOFs film on nylon membrane.

The Zn-HHTP film on nylon membrane was constructed based on a metal-hydroxide-nanostrand-assisted film formation technique. In the first step, A ZIF-8 film with ~500 nm thickness was synthesized on nylon membrane as described previously with some modifications[4]. Typically, equal volumes of a 4 mM $Zn(NO_3)_2 \cdot 6H_2O$ solution and a 2.0 mM aminoethanol solution were mixed rapidly and aged at 25 °C for 30 min. Filtering 30 mL of the mixture solution through a nylon-66 microporous membrane left a white zinc hydroxide nanostrands film on the membrane. Then the zinc hydroxide nanostrands films on the membrane were reacted in 20 ml, 25 mM Hmim ethanol/water solution (1/4, v/v) at room temperature. A pure ZIF-8 thin film typically formed after 24 h at room temperature.

Following the ZIF-8 thin films on nylon-66 microporous membrane was transformed into Zn-HHTP films with different morphologies as described on silicon wafers. Typically, a nylon-66 microporous membrane with ZIF-8 film was reacted with 30 mL of HHTP ethanol/water solution for 24 h. The Zn-HHTP-H films were synthesized by reacting the ZIF-8 film with 1mg/mL solution of HHTP in ethanol/water solution (7:1 v/v) at room temperature. The Zn-HHTP-HS films were synthesized by reacting the ZIF-8 film with 1mg mL^{-1} solution of HHTP in ethanol/water solution (5:3 v/v) at 40 °C. The obtained films were washed with methanol and dried at 80 °C overnight for further use.

The Zn-HHTP-B film was synthesised via directly transformation of the zinc hydroxide nanostrands films on the membrane into Zn-HHTP-B film. Filtering 30 mL of the mixture solution through a nylon-66 microporous membrane left a white zinc hydroxide nanostrands film on the membrane. Then Zn-HHTP-B films were synthesized by reacting the zinc hydroxide nanostrands film directly with 32 mL of 1 mg ml^{-1} HHTP in ethanol/water solution (6:2, v/v) at 70 °C for 24 h. The obtained Zn-HHTP-B films were washed with methanol and dried at 80 °C overnight for further use.

The PcCu-Zn-H film on nylon 66 membrane was constructed with the similar procedure as Zn-HHTP film. Typically, the ZIF-8 film was firstly synthesised on the nylon 66 membrane. The ZIF-8 thin film was transformed into PcCu-Zn-H film with hollow nanostructure as described on silicon wafers. A 28 mL of 1 mg ml^{-1} PcCu-$(OH)_8$ solution and 120 mg sodium acetate in DMF/water solution (7:1, v/v) was prepared firstly. Followed a nylon-66 microporous membrane with ZIF-8 film was immersed into the solution and reacted at room temperature for 24 h. The obtained PcCu-Zn-H films were washed with methanol and dried at 80 °C overnight for further use.

The PcCu-Zn-B film was synthesised via directly transformation of the zinc hydroxide nanostrands films on the membrane into PcCu-Zn-B film. Filtering 30 mL of the mixture solution through a nylon-66 microporous membrane left a white zinc hydroxide nanostrands film on the membrane. Then PcCu-Zn-B films were synthesized by reacting the zinc hydroxide nanostrands film with 32 mL of 1 mg ml^{-1} PcCu-$(OH)_8$ in DMF/water solution (6:2, v/v) at 85 °C for 24 h. The obtained Zn-HHTP-B films were washed with methanol and dried at 80 °C overnight for further use.

The Co-HHTP film on nylon membrane was also constructed based on a metal-hydroxide-nanostrand-assisted film formation technique. In the first step, a ZIF-67 film with ~500 nm thickness was synthesized on nylon membrane as described previously with some modifications[4]. Typically, Equal volumes of a 4 mM $Co(NO_3)_2 \cdot 6H_2O$ solution and a 2.0 mM aminoethanol solution were mixed rapidly and aged at 25 °C for 30. Filtering 30 mL of the mixture solution through a nylon-66 microporous membrane left a green cobalt hydroxide nanostrands film on the membrane. Then the cobalt hydroxide nanostrands films on the membrane were reacted in 20 ml, 0.6 M Hmim ethanol/water solution with volume ratio of 1:4 at room temperature. A purple ZIF-67 thin film typically formed after 24 h at room temperature.

Following the ZIF-67 thin films on nylon-66 microporous membrane was transformed into Co-HHTP films with different morphologies as described on silicon wafers.

Typically, a nylon-66 microporous membrane with ZIF-67 film was reacted with 30 mL of HHTP ethanol/water solution for 24 h. The Zn-HHTP-H films were synthesized by reacting the ZIF-67 film with 1mg mL^{-1} solution of HHTP in ethanol/water solution (7:1 v/v) at room temperature. The obtained films were washed with methanol and dried at 80 °C overnight for further use.

The Zn-HHTP-B film was synthesised via directly transformation of the zinc hydroxide nanostrands films on the membrane into Zn-HHTP-B film. Filtering 30 mL of the mixture solution through a nylon-66 microporous membrane left a white zinc hydroxide nanostrands film on the membrane. Then Zn-HHTP-B films were synthesized by reacting the zinc hydroxide nanostrands film with 32 mL of 1 mg ml^{-1} HHTP in ethanol/water solution (6:2, v/v) at 25 °C for 24 h. The obtained Zn-HHTP-B films were washed with methanol and dried at 80 °C overnight for further use.

Gas concentration regulation for gas sensing

The volume of ammonia water for gas concentration regulation was described as follows:

$$V_x = \frac{VCM}{22.4 \times DP} \times 10^{-9} \times \frac{273 + T_R}{273 + T_B} \qquad (S3)$$

Where, V is the volume of the gas sensing test chamber, which is 20 L; C is the gas concentration, ppm; M is liquid molecular weight; D is the liquid density, g cm^{-3}; P is the liquid purity; T_R is the room temperature, °C; T_B is the temperature inside the gas sensor test chamber, °C; V_x is the volume of fluid to be injected.

Taking 50 ppm ammonia gas as an example, V_x is calculated to be 3.2 uL according to the above formula.

Using a micro-syringe to take a certain volume of ammonia water and put it into a high-temperature evaporating dish (150 °C) inside the gas-sensing test chamber. Then ammonia water will evaporate into ammonia gas. Under the continuous agitation of the fan, the ammonia gas quickly would fill the entire test chamber and diffuse to the surface of the sensor. The overall gas flow rate defaults to 20 L min^{-1}.

Analysis of sensing data.

Here, the sensing response is denoted as Equation S4, and the response/recovery time is denoted as the total resistance change time (from 0 to 90%) at which the sensor

adsorbs and desorbs the target gas.

Raw current data (collected under constant applied voltage of 1.0 V) was normalized and converted to normalized conductance according to Equation S3, wherein I_0 = initial current and I = current at various points during measurement.

$$\frac{\Delta G}{G_0} = \frac{I - I_0}{I_0} \times 100\% \qquad (S4)$$

The theoretical limits of detection (LOD) were calculated using reported protocols[5-7]. First, the root mean squared (rms) value - representing the noise-based deviation in $\Delta G/G_0$ — was calculated using the baseline trace before exposure to analyte. We took 600-1000 consecutive points (N) and fit the data to a polynomial (5th order). We then calculated sum of squared residuals (SSR) from Equation S5, where y_i is measured $\Delta G/G_0$ and y is the value calculated from the polynomial fit. The root-mean-square deviation (RMS) was then calculated by Equation S6. We plotted concentration of analyte versus response ($\Delta G/G_0$) after a specific exposure time and isolated the range of values wherein this relationship was linear. Linear regression provided an equation of best-fit (slope = m). With these values, we extrapolated the theoretical LOD from Equation S7.

$$\mathrm{SSR} = \sum (y_i - y)^2 \qquad (S5)$$

$$\mathrm{RMS} = \sqrt{\frac{\mathrm{SSR}}{N}} \qquad (S6)$$

$$\mathrm{LOD} = 3 \times \frac{\mathrm{RMS}}{m} \qquad (S7)$$

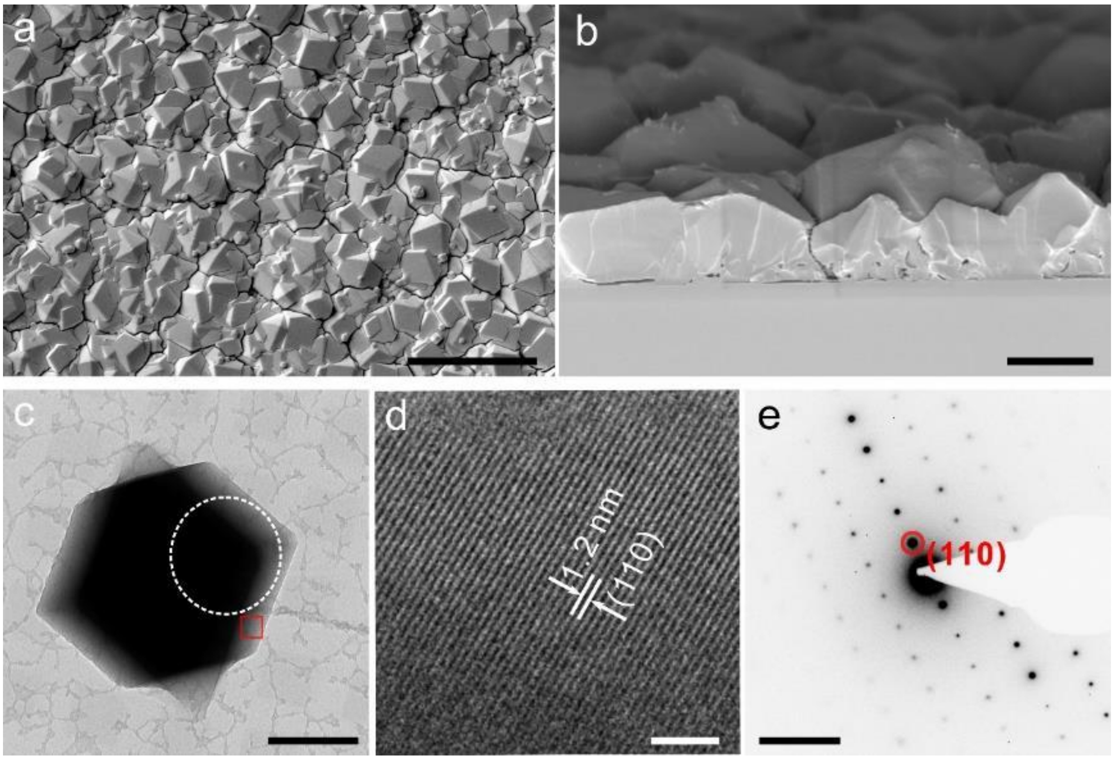

Supplementary Figure 1. (a，b) Surface and cross-section SEM images of the ZIF-8 thin film precursor. (c) TEM image of the ZIF-8 nanoparticle. (d) High-resolution TEM image of the red square shown in (c). (e) The SAED pattern (white circle in (c)). Scare bars were 2 μm for (a), 500 nm for (b), 200 nm for (c), 10 nm for (d) and 2 1/nm for (e).

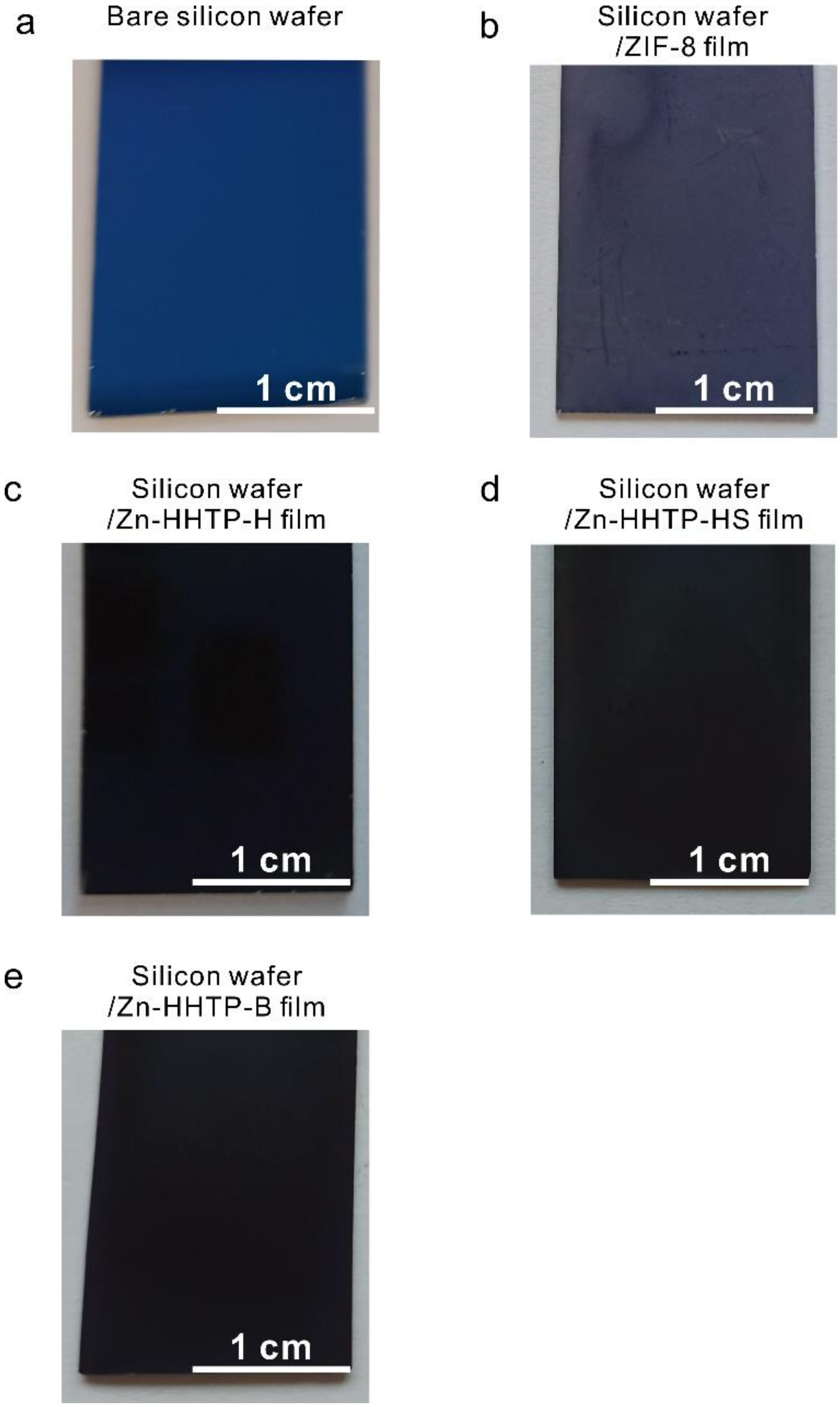

Supplementary Figure 2. (a) Photograph of bare Si/SiO_2 wafer, (b) Photograph of ZIF-8 film coated Si/SiO_2 wafer, (c) Photograph of as-synthesized Zn-HHTP-H film coated Si/SiO_2 wafer, (d) Photograph of as-synthesized Zn-HHTP-HS film coated Si/SiO_2 wafer. (e) Photograph of as-synthesized Zn-HHTP-B film coated Si/SiO_2 wafer.

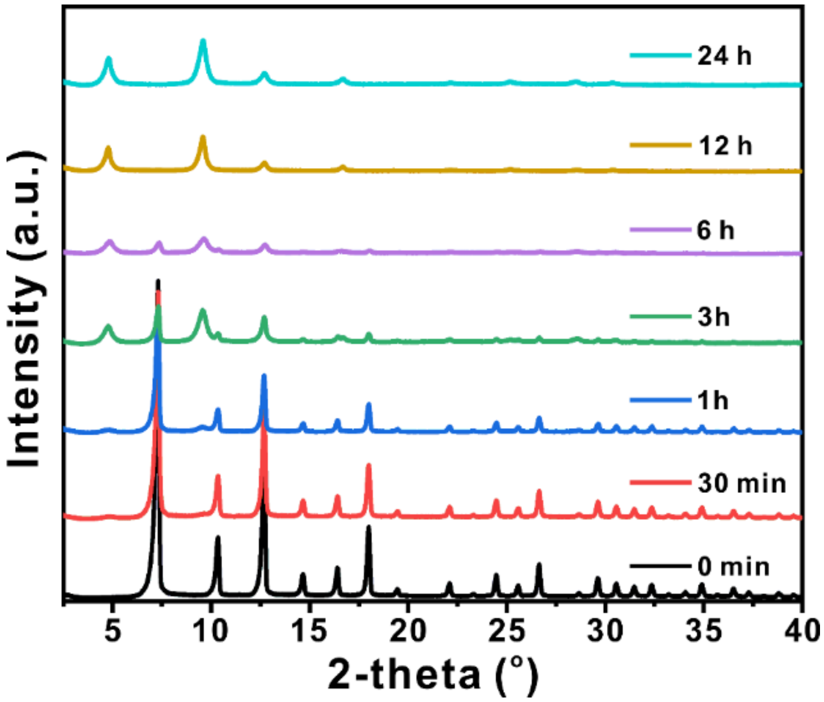

Supplementary Figure 3. Powder XRD patterns of the Zn-HHTP-H products collected at different reaction time.

During the transformation process, the MOFs crystal structure transformation from pure ZIF-8 to Zn-HHTP-H was confirmed by PXRD measurements. In the initial time, PXRD pattern of as-obtained MOFs film matches well with that of crystalline ZIF-8. Then the mixed phase of ZIF-8 and Zn-HHTP was observed at 30 min. With increasing the time to 3 h, the intensity of the diffraction peaks ascribed to Zn-HHTP increased relative to ZIF-8. For the sample at 12 h, the peaks ascribed to ZIF-8 vanished, leaving a single phase of Zn-HHTP. On the basis of the above results, it may be speculated that original and etched rhombic dodecahedron exhibit the ZIF-8 crystal structure, while subsequently formed hollow nanostructures have the Zn-HHTP crystal structure.

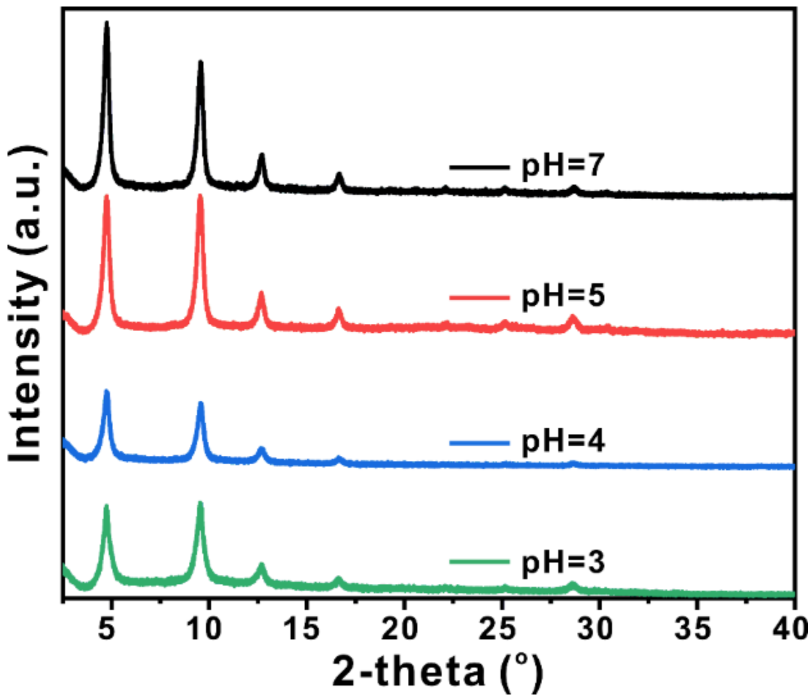

Supplementary Figure 4. Powder XRD patterns of the Zn-HHTP crystals collected at different pH solution.

It is well known that the ZIF-8 can be decomposed at the pH=5 solution[8,9]. However, the Zn-HHTP crystal can maintain its crystal structure at pH=3, suggesting that the ZnO_4 coordination bonds in Zn-HHTP is much more stable than the Zn-N coordination in ZIF-8 crystals.

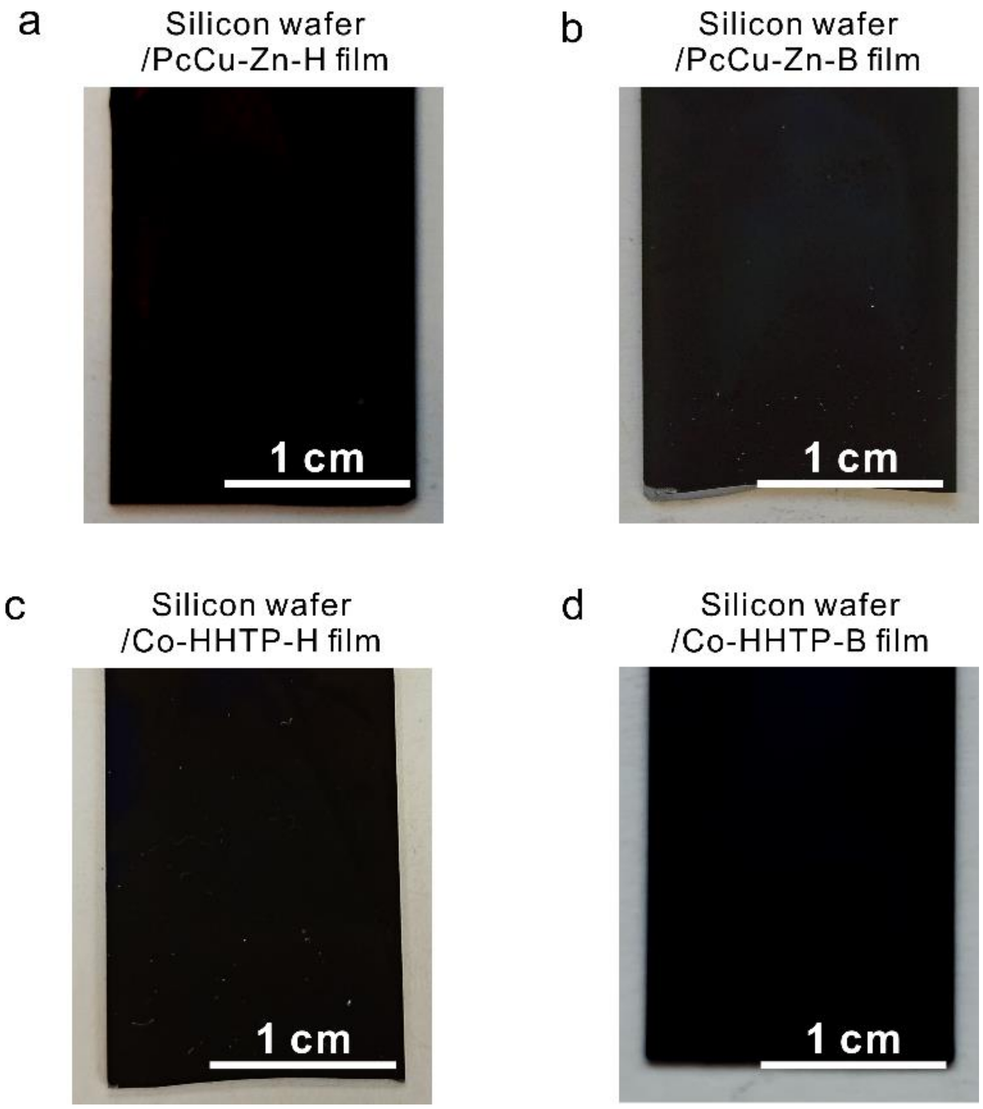

Supplementary Figure 5. (a) Photograph of as-synthesized PcCu-Zn-H film coated Si/SiO_2 wafer. (b) Photograph of as-synthesized PcCu-Zn-B film coated Si/SiO_2 wafer. (c) Photograph of as-synthesized Co-HHTP-H film coated Si/SiO_2 wafer. (d) Photograph of as-synthesized Co-HHTP-B film coated Si/SiO_2 wafer.

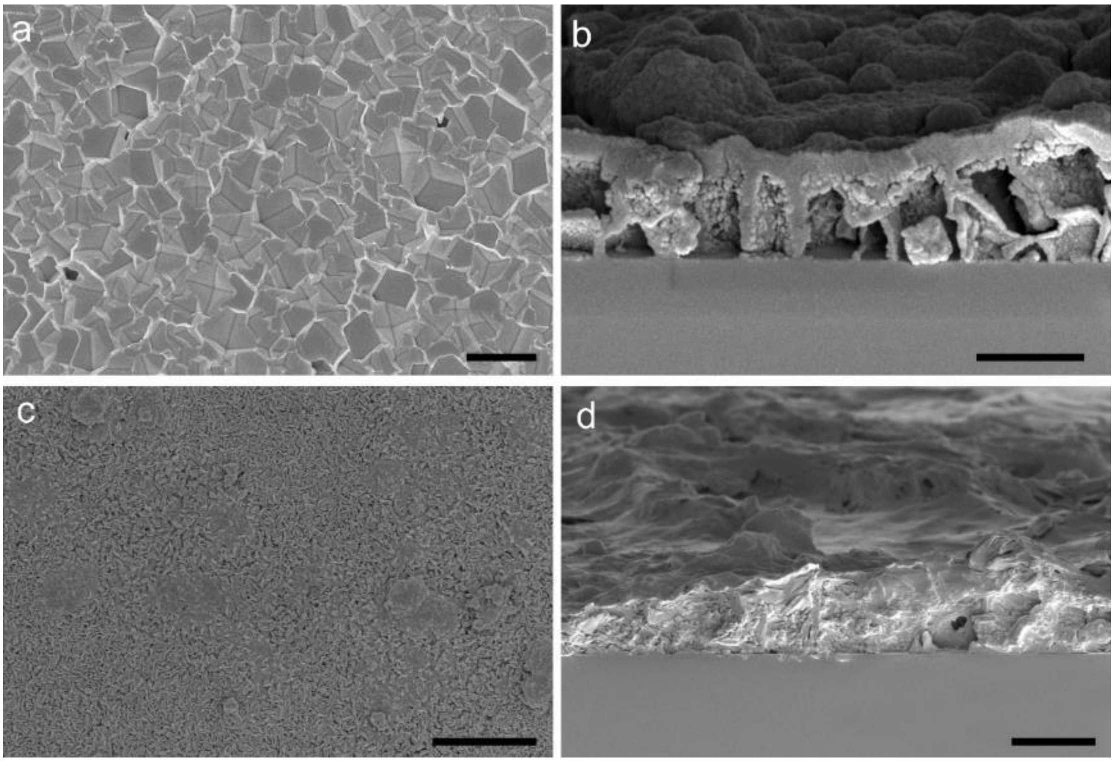

Supplementary Figure 6. (a，b) Surface and cross-section SEM images of the PcCu-Zn-H thin film. (c，d) Surface and cross-section SEM images of the PcCu-Zn-B film. Scare bars were 1 μm for (a) and (c), 500 nm for (b) and (d).

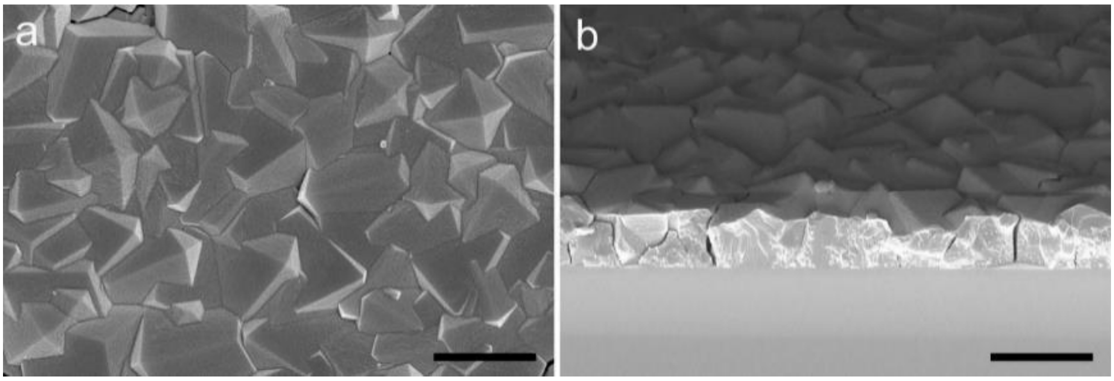

Supplementary Figure 7. (a，b) Surface and cross-section SEM images of the ZIF-67(Co) thin film. Scare bars were 1 μm for (a) and (b).

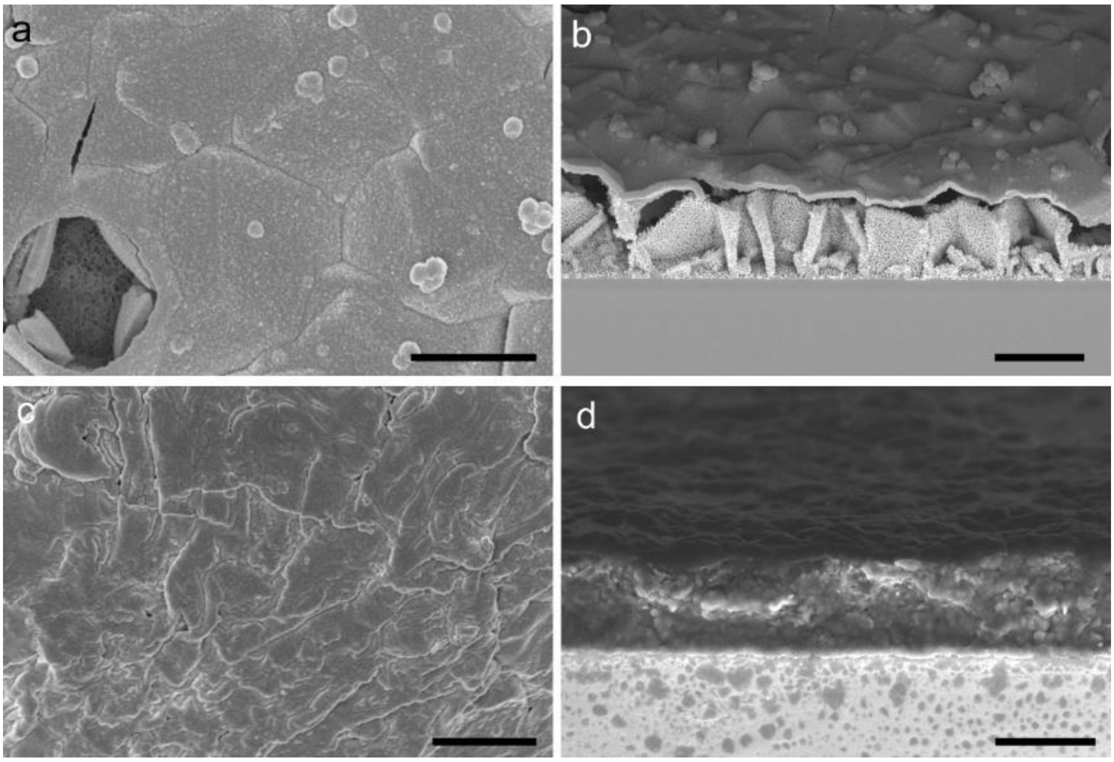

Supplementary Figure 8. (a, b) Surface and cross-section SEM images of the Co-HHTP-H thin film. (c, d) Surface and cross-section SEM images of the Co-HHTP-B film. Scare bars were 1 μm for (a) and (c), 500 nm for (b) and (d).

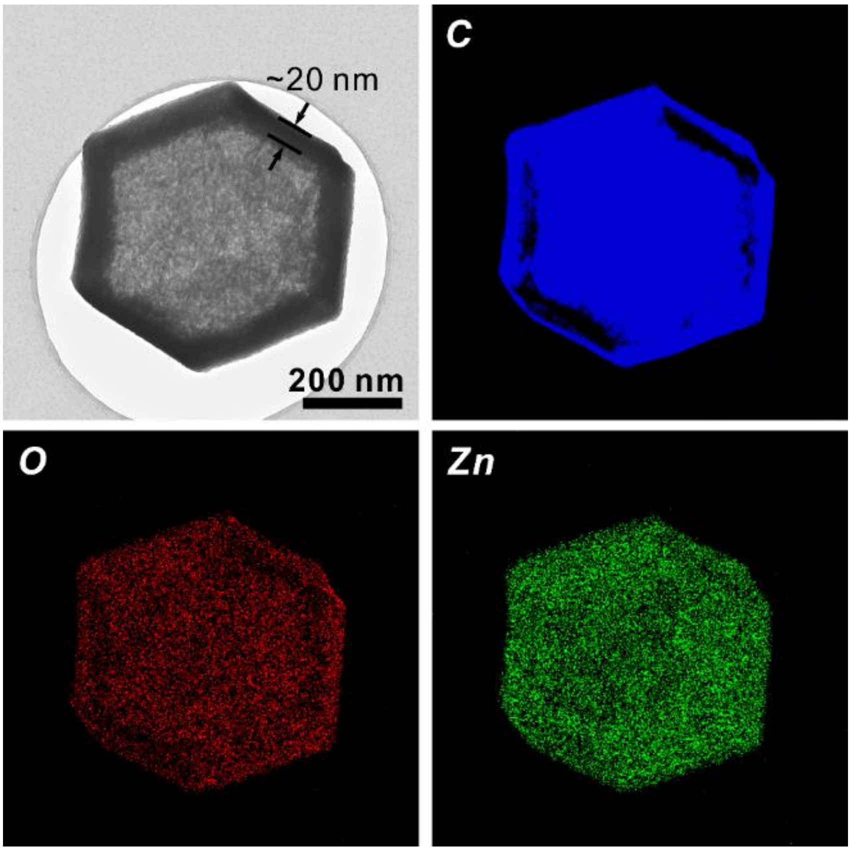

Supplementary Figure 9. TEM image of the Zn-HHTP-H nanoparticle and the corresponding low-loss electron energy loss spectroscopy (EELS) mapping of the Zn-HHTP-H nanoparticle in TEM image. Scale bars, 200 nm.

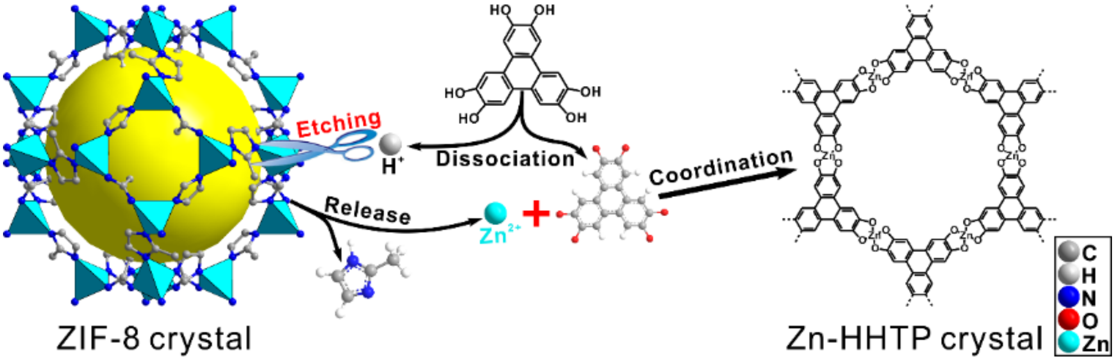

Supplementary Figure 10. Schematic overview of the transformation of ZIF-8 to Zn-HHTP crystal.

The protons dissociated from HHTP ligands acted as reaction trigger to break coordinate bonds between Zn^{2+} and mim^{-} linkers in the ZIF-8 (equations 1 and 2). The surface of ZIF-8 nanocrystal gradually decomposed and the dissolved Zn^{2+} were released into the solution. The free Zn^{2+} ions were coordinated with deprotonated HHTP ligands and deposited onto the surface of ZIF-8 nanocrystals to form a core-shell hybrid structure (equation 3). The time-dependent TEM imaging indicated that, as the ZIF-8 nanocrystals continued to decompose, the gap between the inner ZIF-8 and the Zn-HHTP shell increases while the shell gets thicker. Finally, the inside ZIF-8 crystal disappeared completely, leading to the formation of Zn-HHTP-H with hollow nanostructure (equation 4).

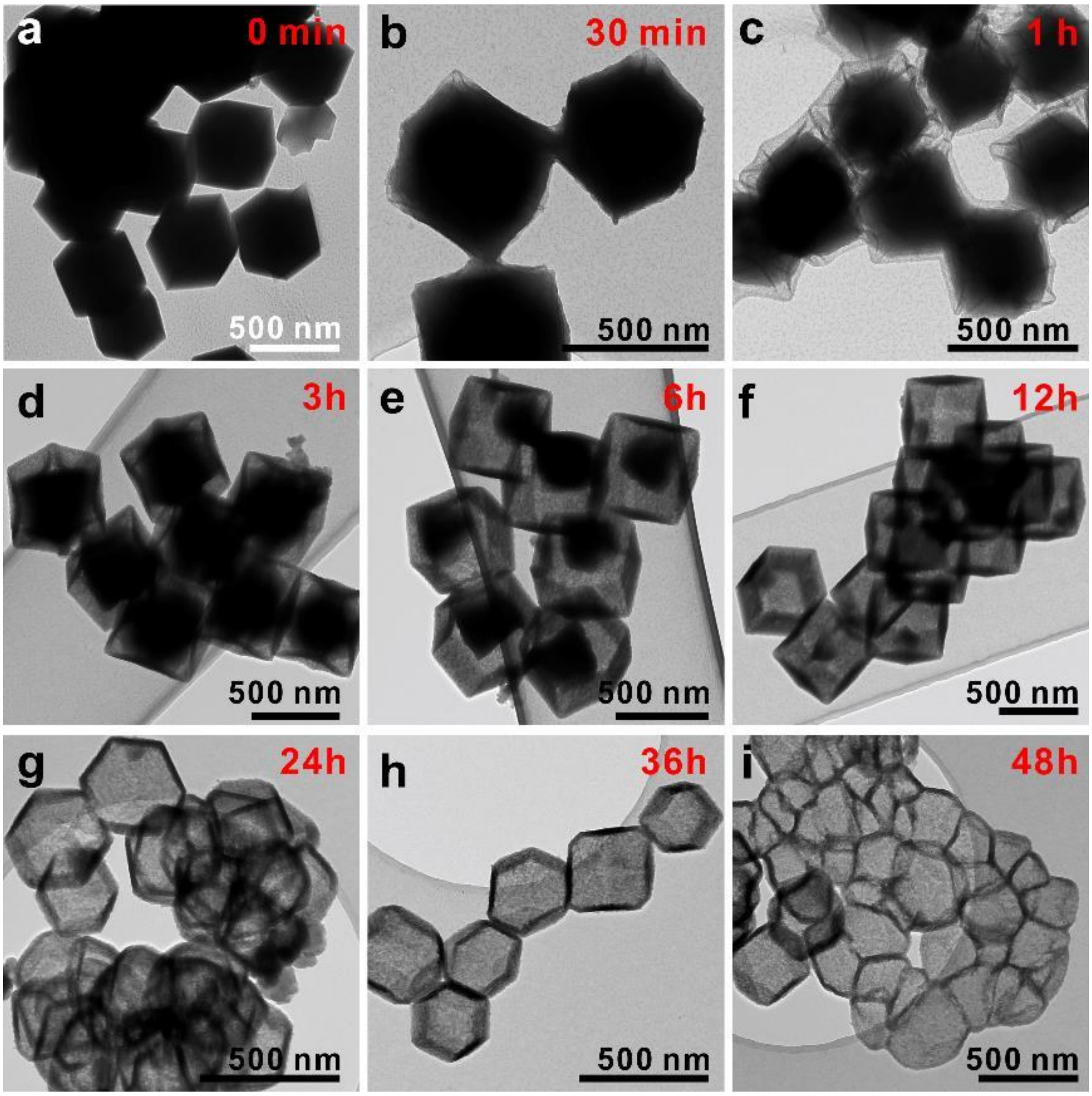

Supplementary Figure 11. (a-i) TEM images of ZIF-8 transformed into Zn-HHTP-H at different reaction time (from 0 min to 48 h). These Zn-HHTP-H was scraped off from the corresponding Zn-HHTP-H films. Scare bars represent 500 nm for (a-i).

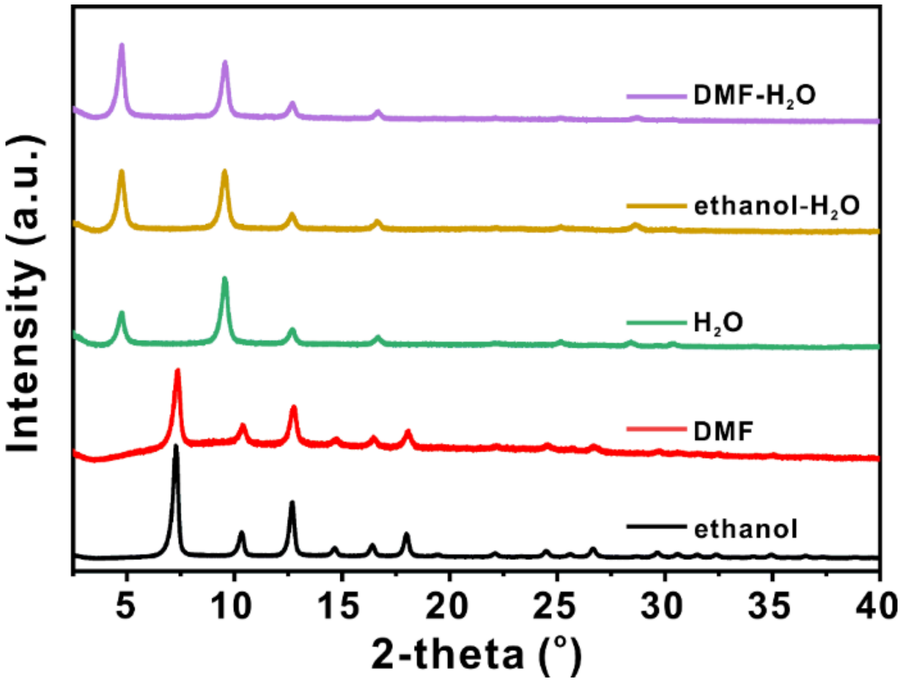

Supplementary Figure 12. Powder XRD patterns of the Zn-HHTP products collected at different reaction solvents.

When only organic solvent was used, no Zn-HHTP product was observed. It was supposed that the generated protons in organic solvent were too less to break the coordination bonds between Zn^{2+} and MeIM linkers in the ZIF-8 crystals. Consequently, the etching step in equation 2 ($3Zn(MeIM)_2 + 6H^+ \rightleftharpoons 6HMeIM + 3Zn^{2+}$) was inhibited and finally no Zn-HHTP product was produced.

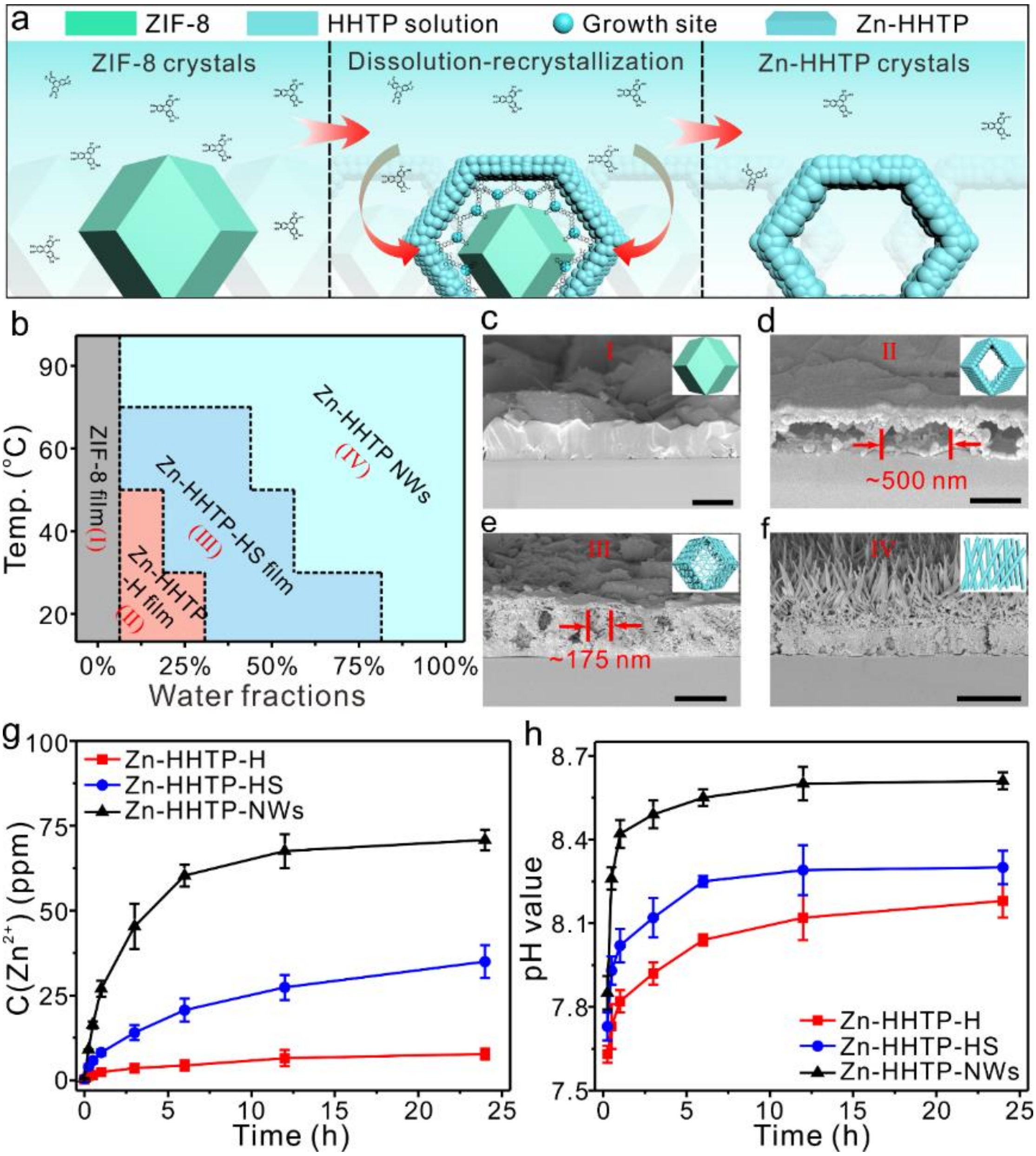

Supplementary Figure 13. (a) Schematic overview of the transformation of ZIF-8 to Zn-HHTP-H film. (b) Phase diagram that correlates the solvent composition (horizontal ordinate) and reaction temperature (vertical ordinate). (c, d, e, f) Cross-sectional SEM images of the ZIF-8, Zn-HHTP-H, Zn-HHTP-HS and Zn-HHTP nanowire (NW) films, respectively. (g, h) The concentration of Zn^{2+} (g) and pH value (h) versus reaction time toward the construction of Zn-HHTP-H, Zn-HHTP-HS and Zn-HHTP NW films, respectively. Scale bars represent 500 nm for (c-f).

Throughout the whole transformation, the product morphology significantly depends on the reaction kinetics (Supplementary Figure 13b). At low water fractions and low temperatures, the etching rate of ZIF-8 is supposed to be slowed down and much lower than the coordination rate. The diffusion distance of free Zn^{2+} ions is insufficient, and nucleation and growth of Zn-HHTP only occurs on the inner wall of the Zn-HHTP shell. When higher water fractions and temperatures are used, the corresponding etching rate increases and is comparable to the coordination rate. In this case, most of the Zn^{2+} ions prefer to form Zn-HHTP inside the shell, while a small part of the Zn^{2+} ions diffuse into

the solution and produce Zn-HHTP nanowires outside the shell. If the water fractions and temperatures are further increased, the etching rate of ZIF-8 would increase dramatically, faster than the Zn-HHTP shell can form. Therefore, Zn^{2+} ions from the etching of the ZIF-8 nanocrystals mostly diffuse into the solution and react with $(HHTP^{*})^{3-}$ ions to form Zn-HHTP nanowires on the surface of the ZIF-8 nanocrystals. The above results underline that the balance between the etching and coordination rate is crucial to achieve a well-defined hollow nanostructure.

In order to verify the above conversion mechanism, the Zn^{2+} concentration and the pH value were monitored over the reaction time towards the construction of different Zn-HHTP products (Figs. S13g-S13h). The initial Zn^{2+} concentrations in the solution are all around 0.15 ppm. In the case of Zn-HHTP NW, this concentration increased rapidly as the reaction was initiated, revealing the rapid generation of free Zn^{2+} ions in the reactions (Equation 2). Throughout the conversion, the Zn^{2+} concentration continues to increase, reaching approximately 70.0 ppm at equilibrium. For the case of Zn-HHTP-H, the Zn^{2+} concentration shows a very slow growth and is only about 7.7 ppm at equilibrium, which is much lower than those for Zn-HHTP-HS and Zn-HHTP NW. These results indicate that all the conversion for Zn-HHTP-H takes place within the Zn-HHTP shell. However, the nucleation and growth of Zn-HHTP NW takes place outside the surface of ZIF-8 with most of the metal ions being released into the solution. To test the change of the H^{+} concentration, the pH values were investigated (Supplementary Figure 13h). The initial pH of the HHTP solution was only about 5.3 and the pH values were found to increase gradually throughout the transformation. These results suggest that the protons generated in Equation 1 are rapidly consumed in Equation 2, a trend that is highly consistent with the change in Zn^{2+} concentration.

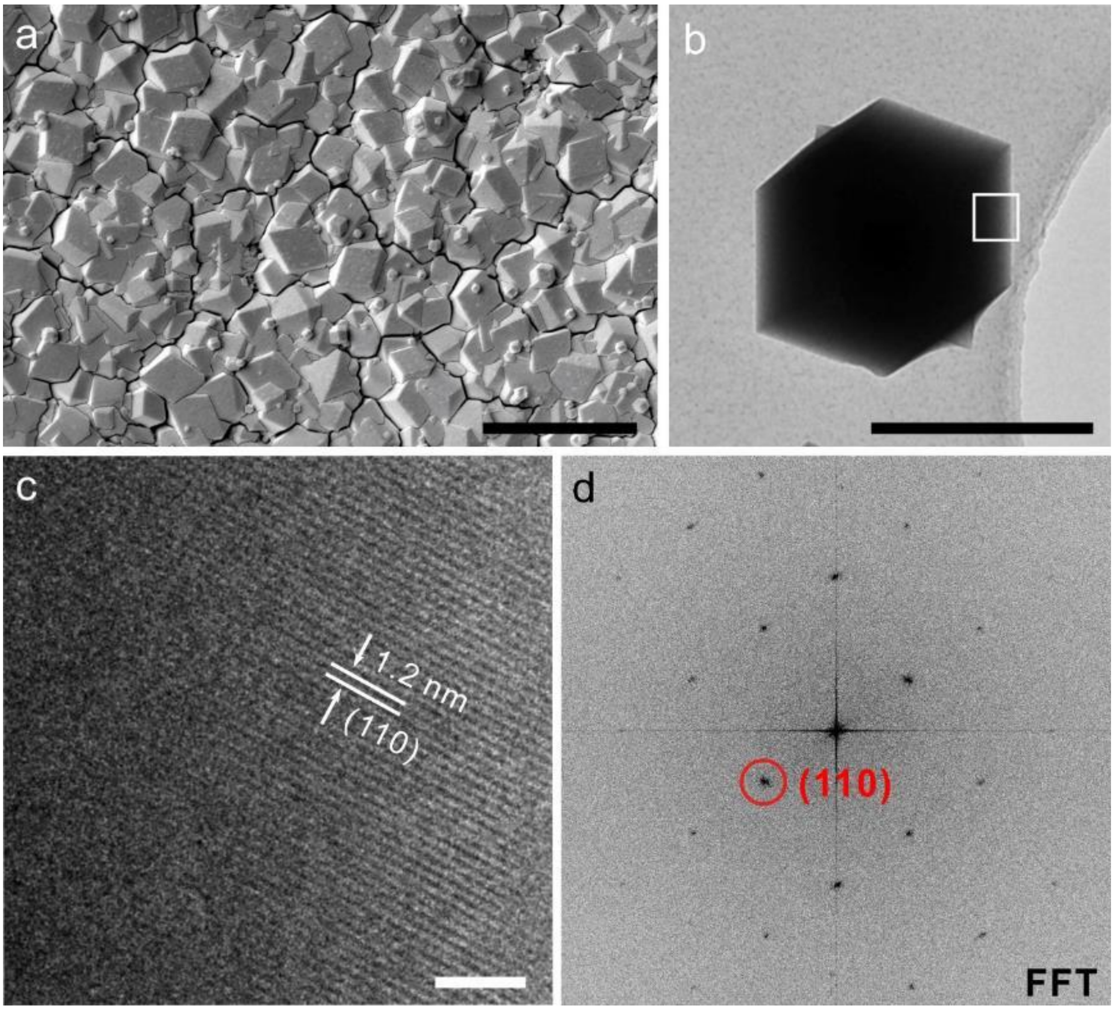

Supplementary Figure 14. (a，b) Surface SEM image of the film product while only organic solvent (ethanol) was used as solvent. (b) TEM image of the ZIF-8 nanoparticle in zone I. (c) High-resolution TEM image of the red square shown in (c). (d) Corresponding FFT image for (c). Scare bars were 2 μm for (a), 500 nm for (b), 10 nm for (c).

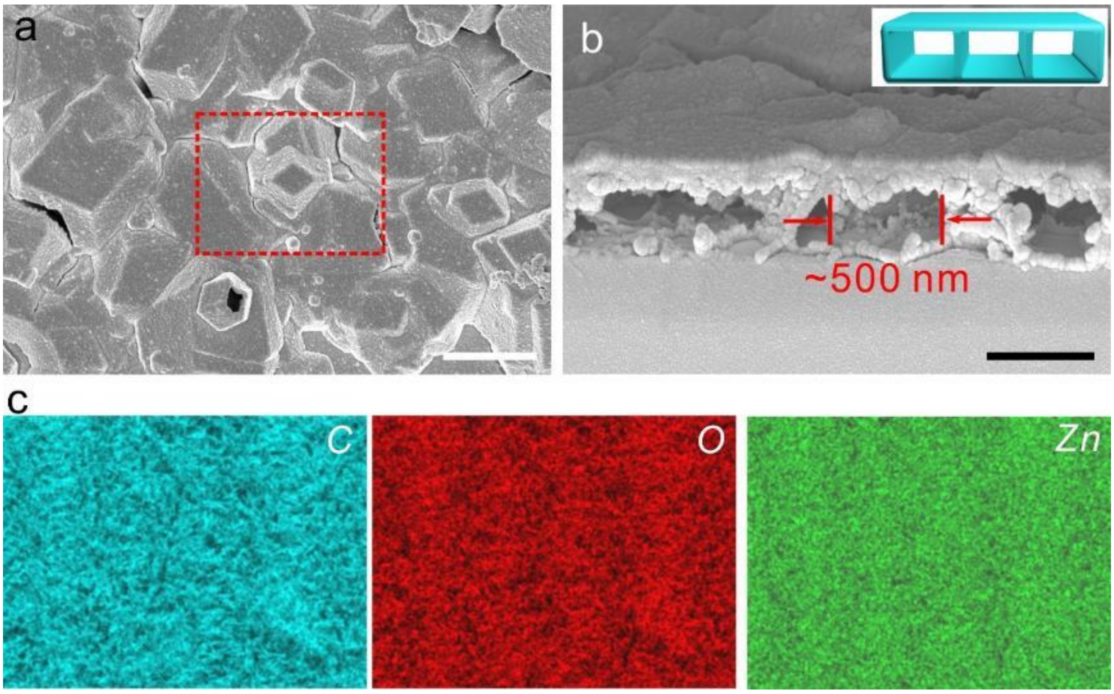

Supplementary Figure 15. (a-b) SEM images of Zn-HHTP-H films in zone II and (c) the energy dispersive X-ray elemental mapping results. Scale bars, 500 nm for (a) and (b).

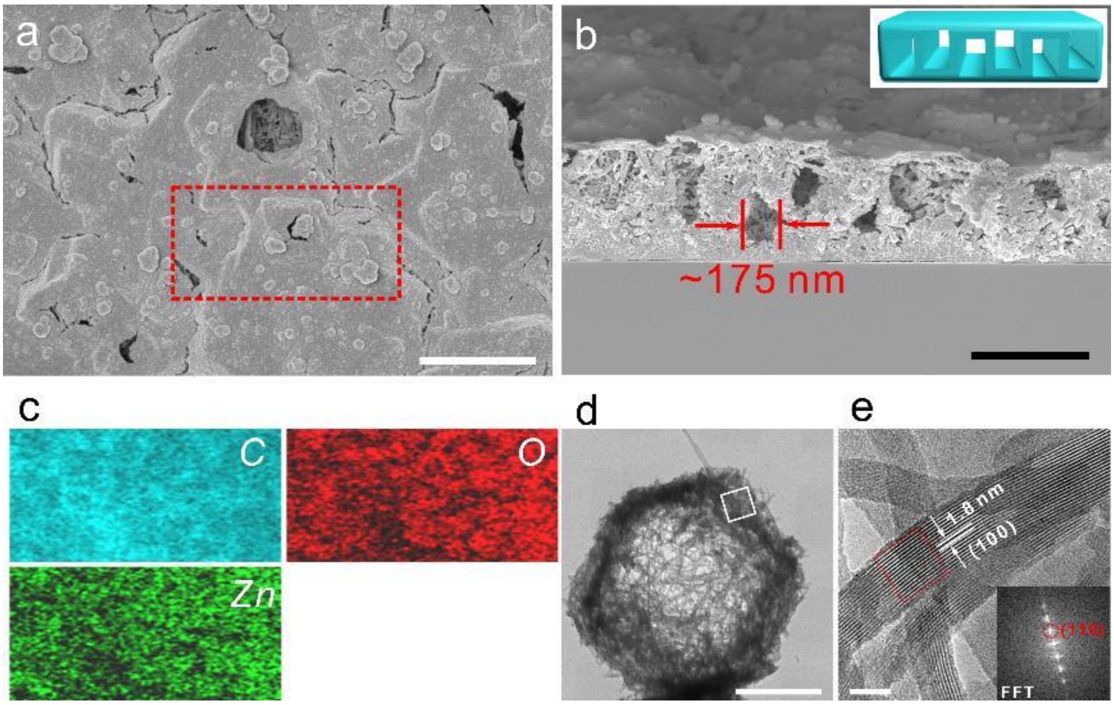

Supplementary Figure 16. (a-b) SEM images of Zn-HHTP-HS films in zone III and (c) the energy dispersive X-ray elemental mapping results. (d) TEM image of the Zn-HHTP-HS nanoparticle. (e) HRTEM image of the of the white square shown in (d). The inset is the FFT image from the square shown in (e). Scale bars, 500 nm for (a) and (b), 200 nm for (d) and 20 nm for (e).

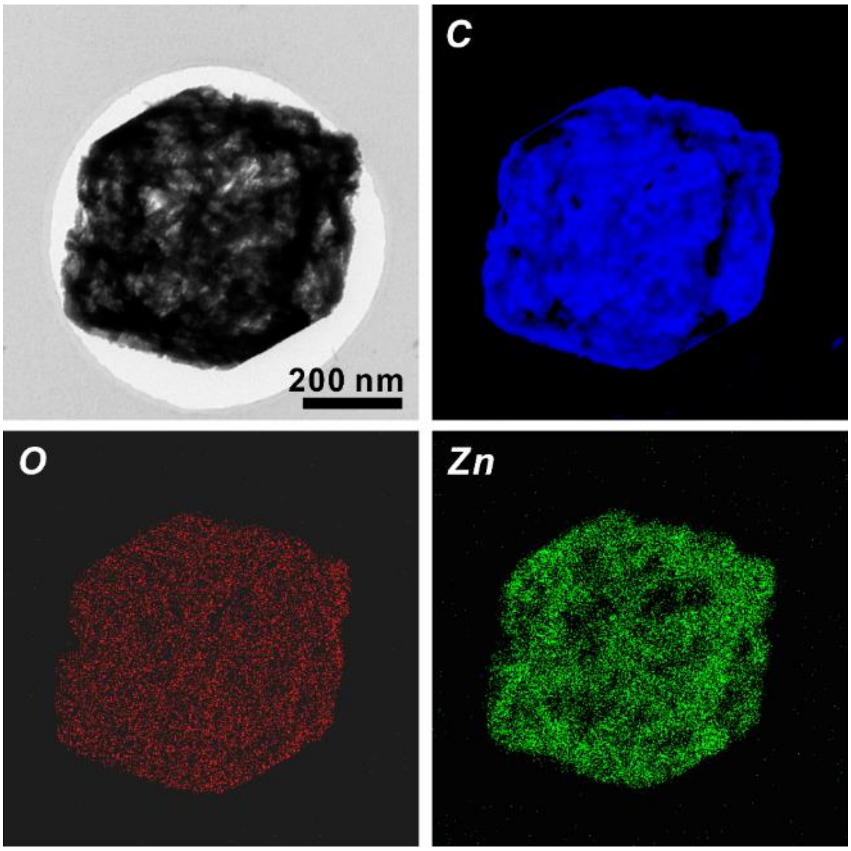

Supplementary Figure 17. TEM image of the Zn-HHTP-HS product and the corresponding EELS mapping of the Zn-HHTP-HS product in TEM image. Scale bars, 200 nm.

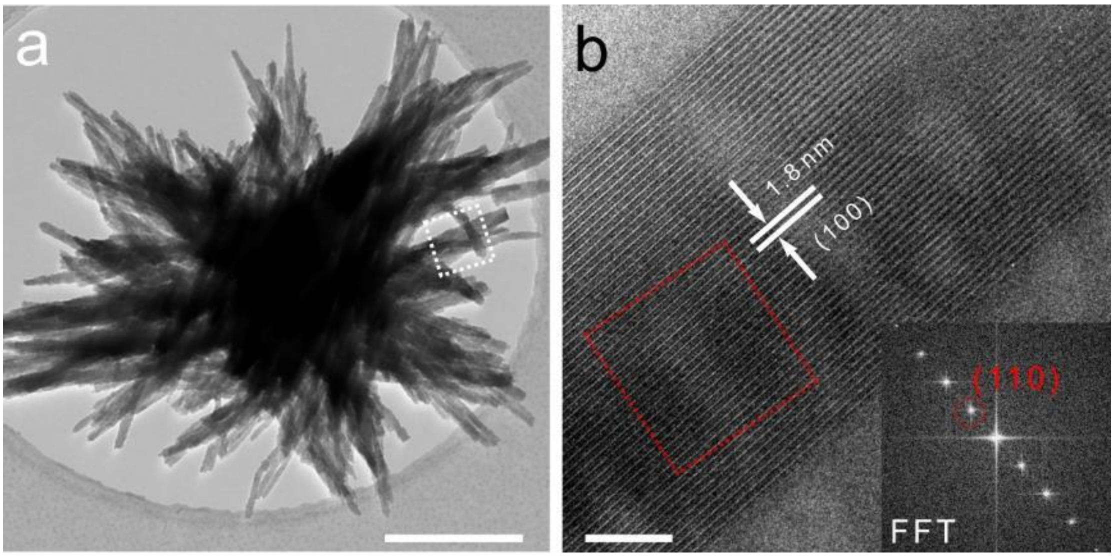

Supplementary Figure 18. (a) TEM image of the Zn-HHTP NWs products. (b) HRTEM image of the of the white square shown in (a). The inset is the FFT image from the square shown in (b). Scale bars, 1 μm for (a) and 20 nm for (b).

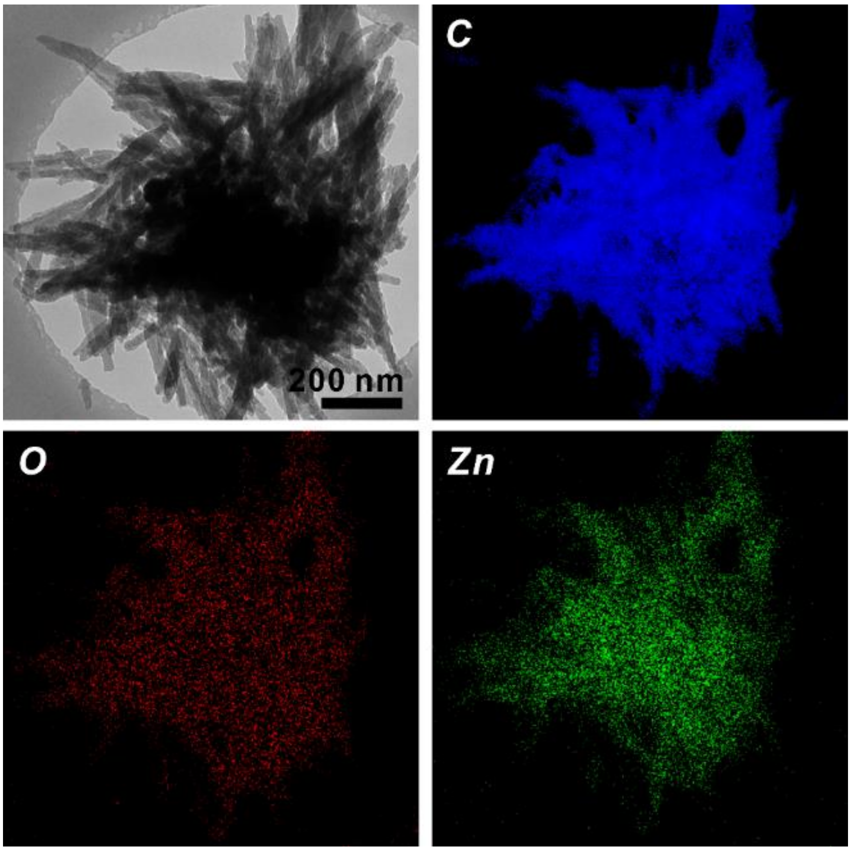

Supplementary Figure 19. TEM image of the Zn-HHTP NWs product and the corresponding EELS mapping of the Zn-HHTP NWs product in TEM image. Scale bars, 200 nm.

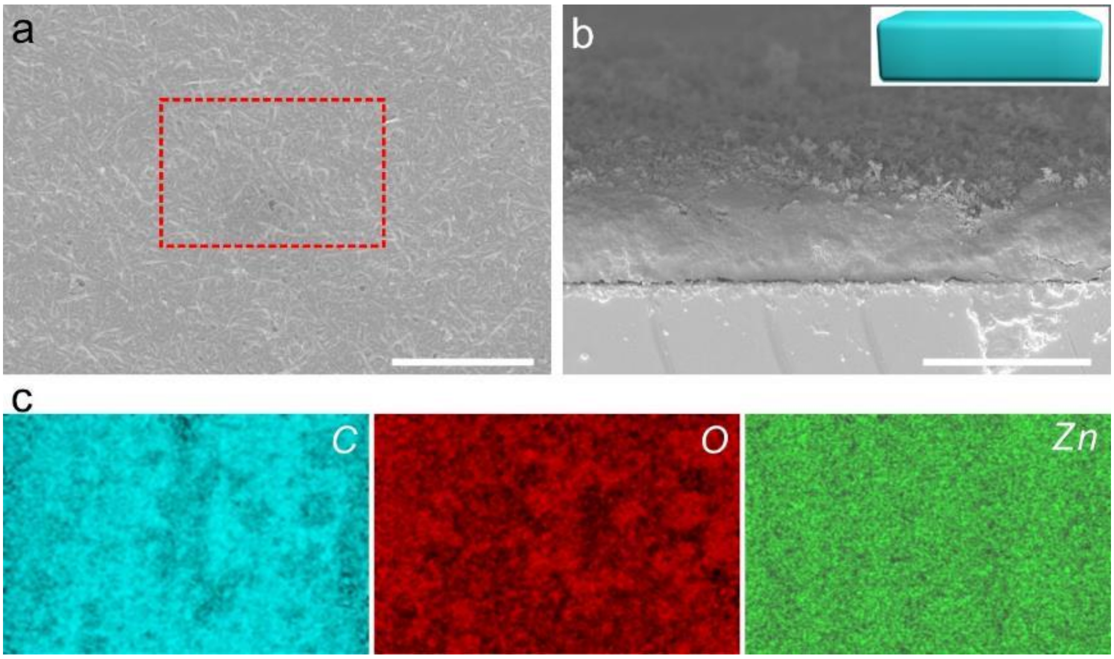

Supplementary Figure 20. (a-b) Surface and cross-section SEM images of Zn-HHTP-B film. (c) The energy dispersive X-ray elemental mapping results for Zn-HHTP-B film. Scare bars represent 2 μm for (a) and 1 μm for (b).

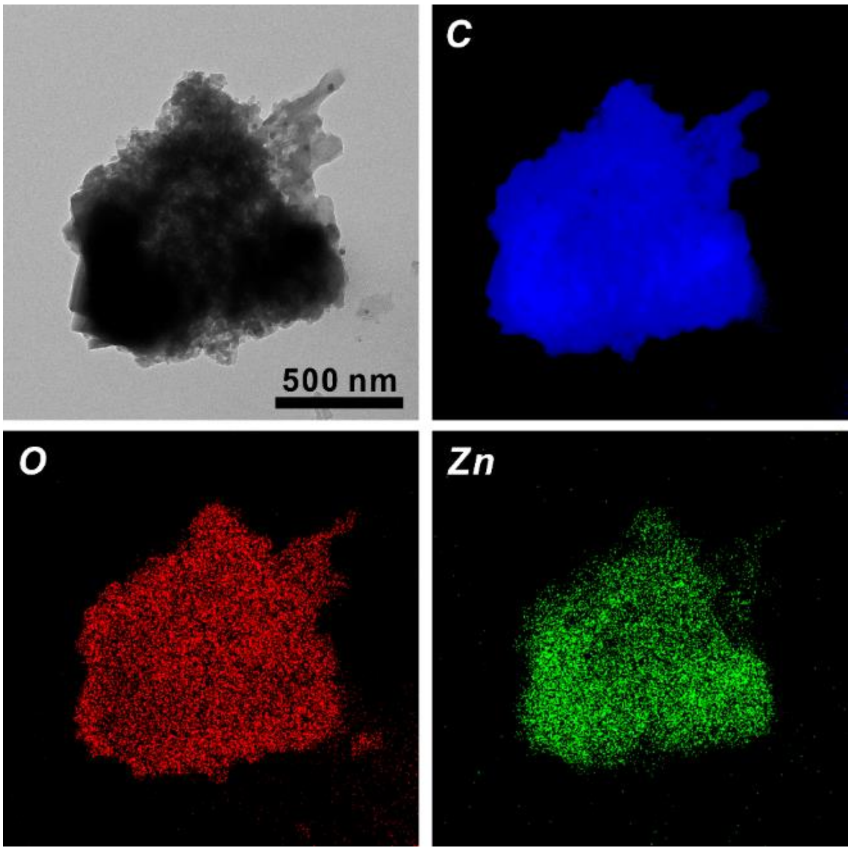

Supplementary Figure 21. TEM image of the Zn-HHTP-B product and the corresponding EELS mapping of the Zn-HHTP-B product in TEM image. Scale bar, 500 nm.

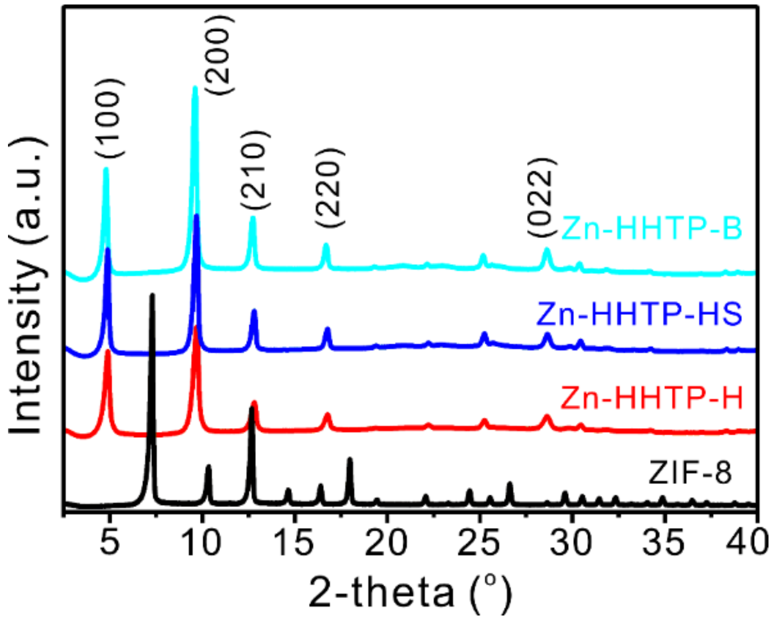

Supplementary Figure 22. Powder XRD patterns of the Zn-HHTP products.

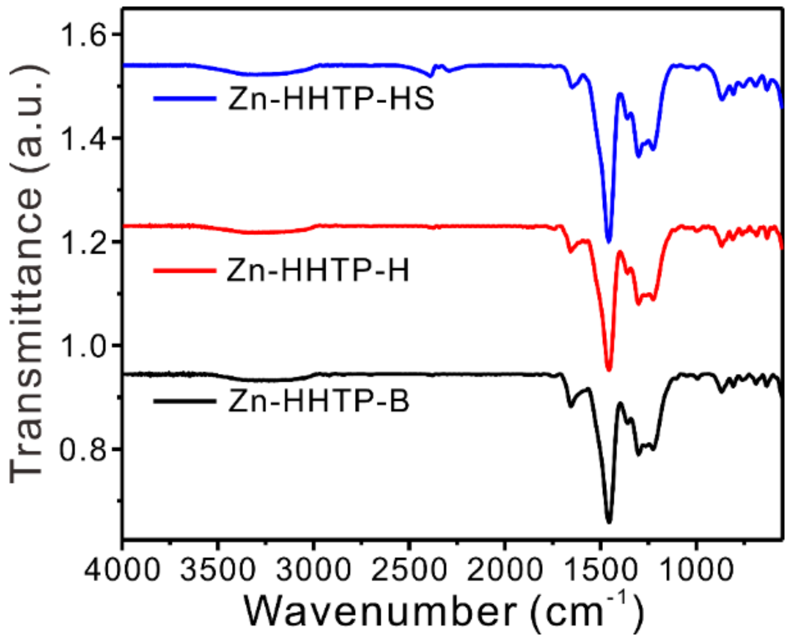

Supplementary Figure 23. FT-IR spectra of as-synthesized Zn-HHTP products.

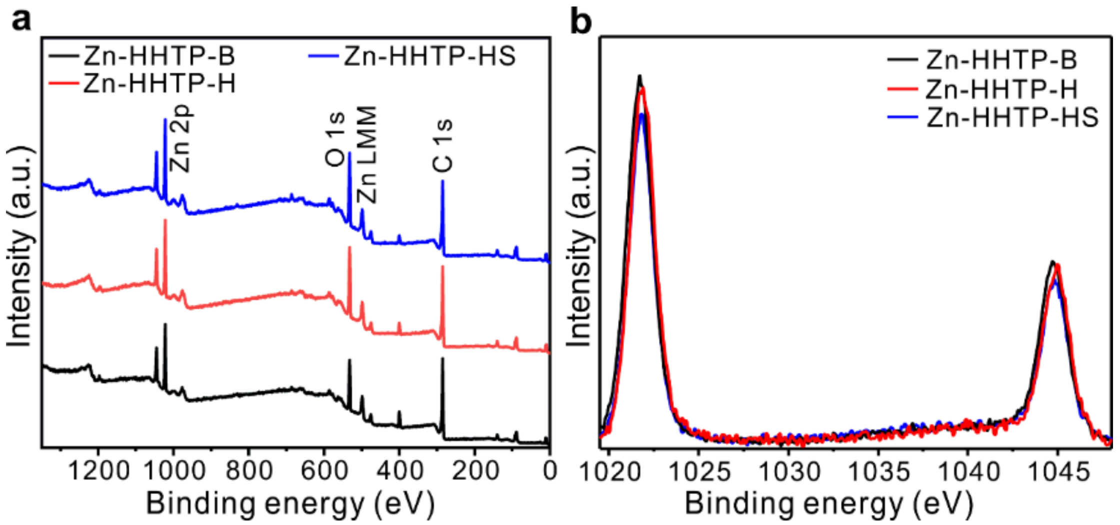

Supplementary Figure 24. (a) XPS patterns of as-synthesized Zn-HHTP products. (b) High-resolution XPS spectra of Zn 2p of as-synthesized Zn-HHTP products.

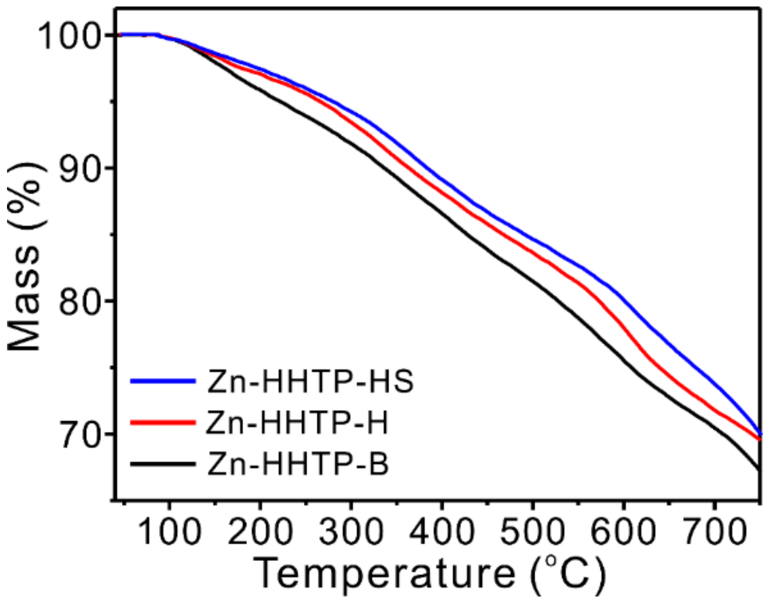

Supplementary Figure 25. TGA curves of as-synthesized Zn-HHTP products.

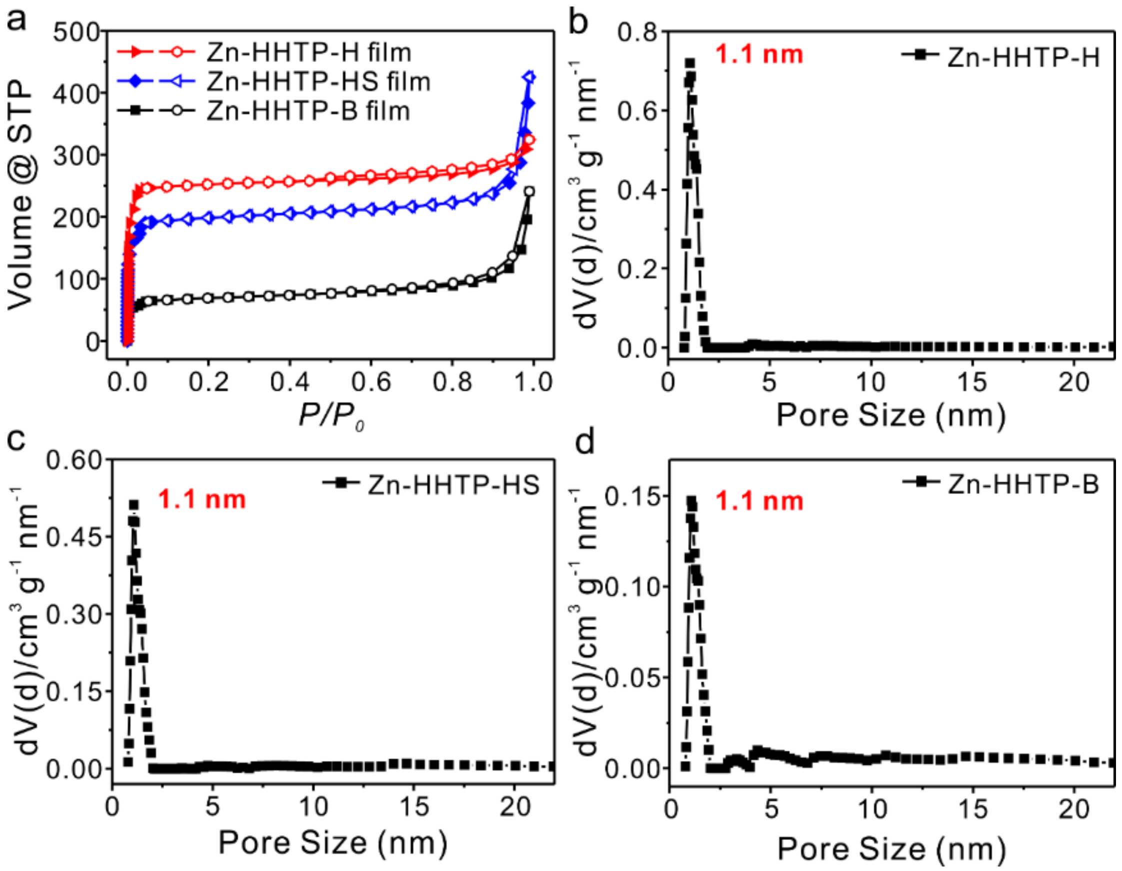

Supplementary Figure 26. (a) Nitrogen adsorption and desorption isotherms measured at 77 K of as-synthesized Zn-HHTP products. (b-d) The corresponding pore size distributions of the Zn-HHTP products.

The BET surface area of Zn-HHTP-H, Zn-HHTP-HS and Zn-HHTP-B films are 614.0, 479.4 and 165.3 m^2 g^{-1}, respectively. The pore volume of Zn-HHTP-H, Zn-HHTP-HS and Zn-HHTP-B films are 0.405, 0.384 and 0.193 cm^3 g^{-1}, respectively.

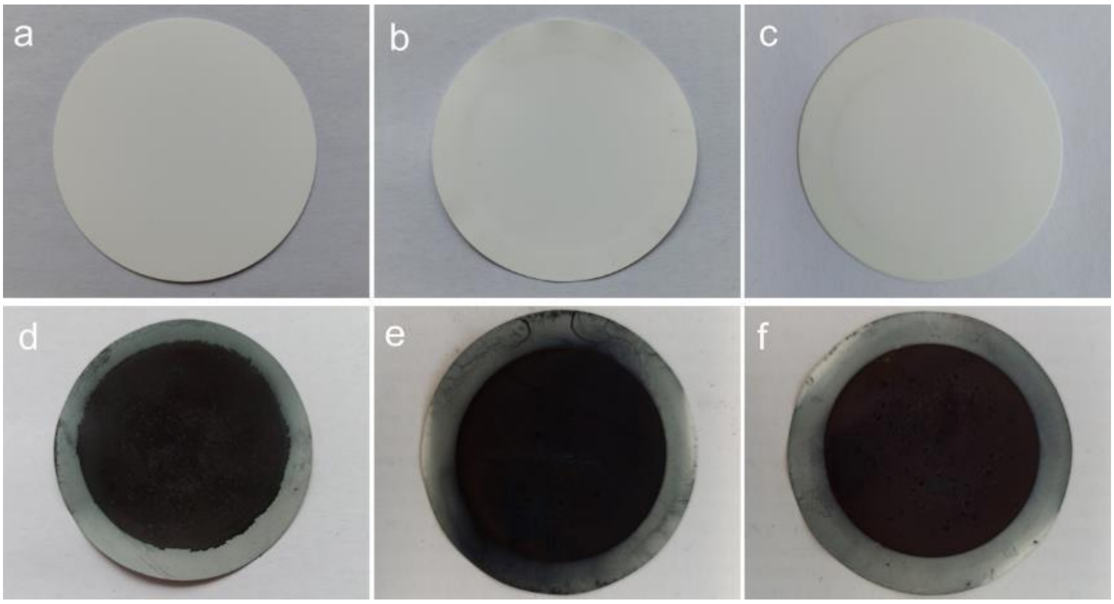

Supplementary Figure 27. (a-c) Photographs of bare nylon 66 membrane, zinc hydroxide nanostrands coated nylon 66 membrane and ZIF-8 thin film coated nylon 66 membrane, respectively. (d-f) Photographs of Zn-HHTP-B, Zn-HHTP-HS and Zn-HHTP-H coated nylon 66 membrane, respectively. The diameter and pore size of nylon 66 membrane are 47.0 mm and 0.22 μm, respectively.

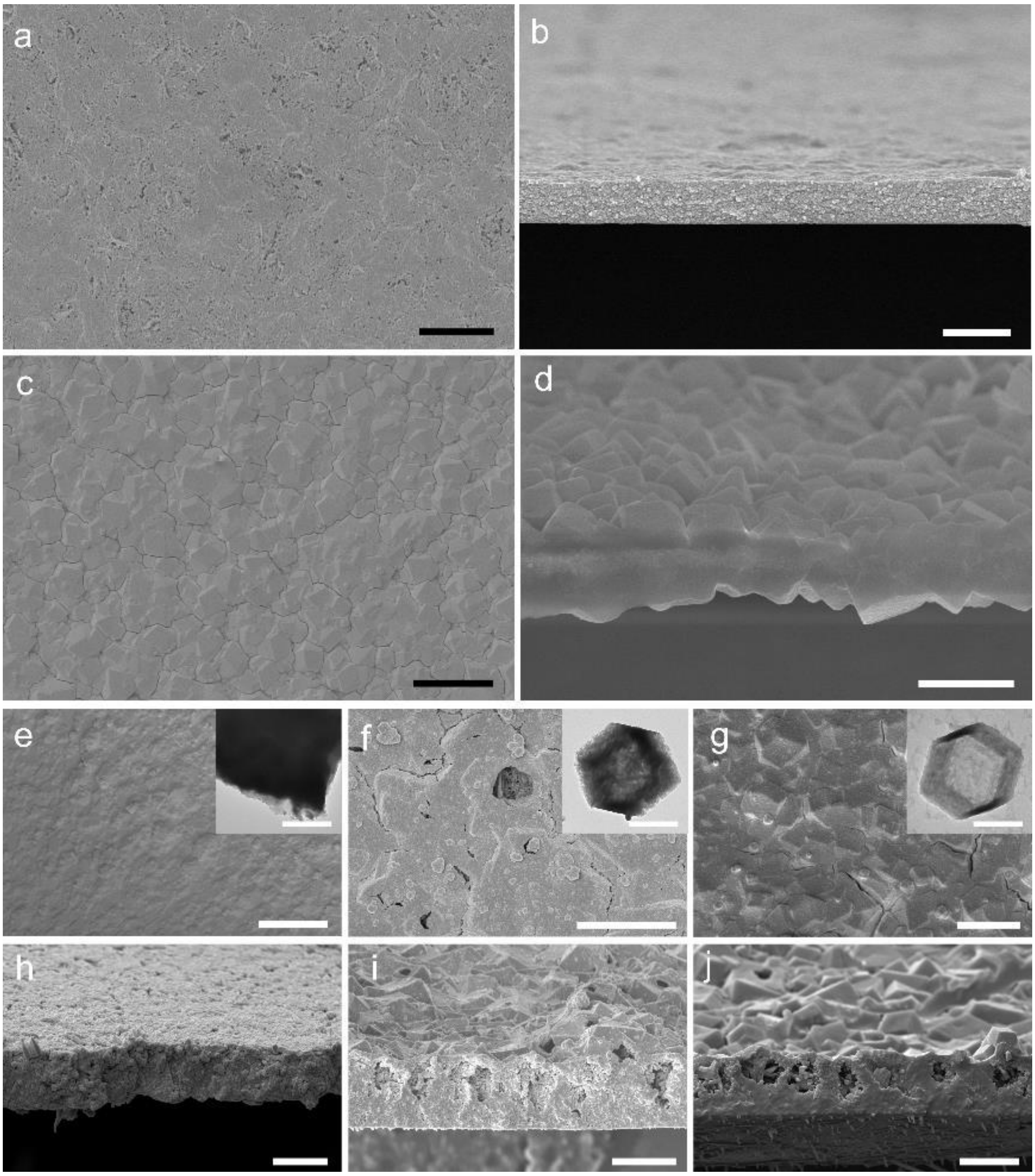

Supplementary Figure 28. (a-b) Surface and cross-section SEM images of the zinc hydroxide nanostrands thin film on nylon 66 membrane. (c-d) Surface and cross-section SEM images of the ZIF-8 thin film on nylon 66 membrane. (e-j) Surface and cross-section SEM images of the Zn-HHTP-B, Zn-HHTP-HS and Zn-HHTP-H films on nylon 66 membrane. The insets in e, f and g are the TEM image Zn-HHTP-B, Zn-HHTP-HS and Zn-HHTP-H nanoparticles, respectively. Scare bars were 1 μm for (a), 500 nm for (b), 1 μm for (c), 500 nm for (d), 500 nm for (e-j), 500 nm for the insets in e, 200 nm for insets in f and g.

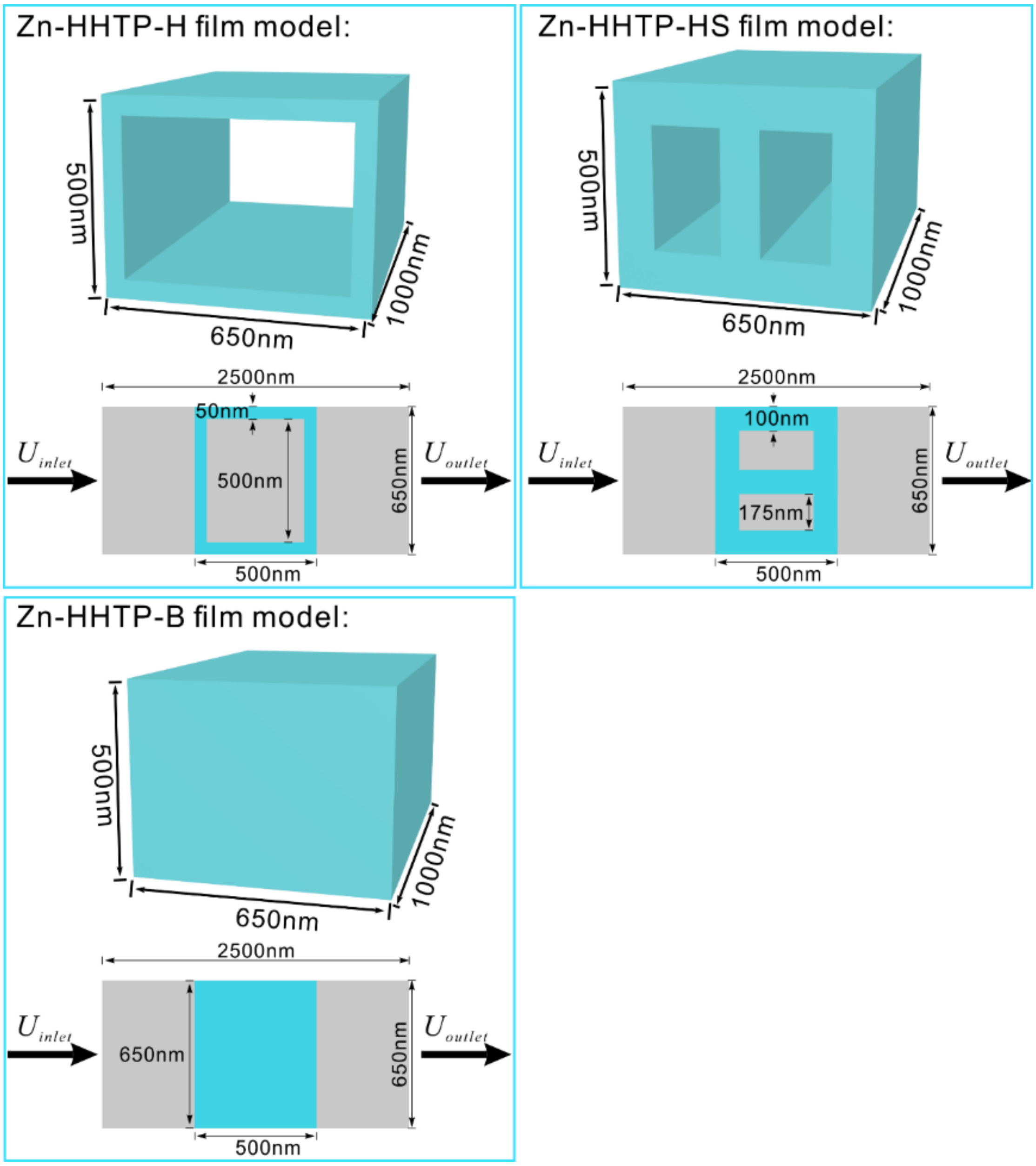

Supplementary Figure 29. Simulation models setup for theoretical pressure-driven flow past the Zn-HHTP porous media in a square channel.

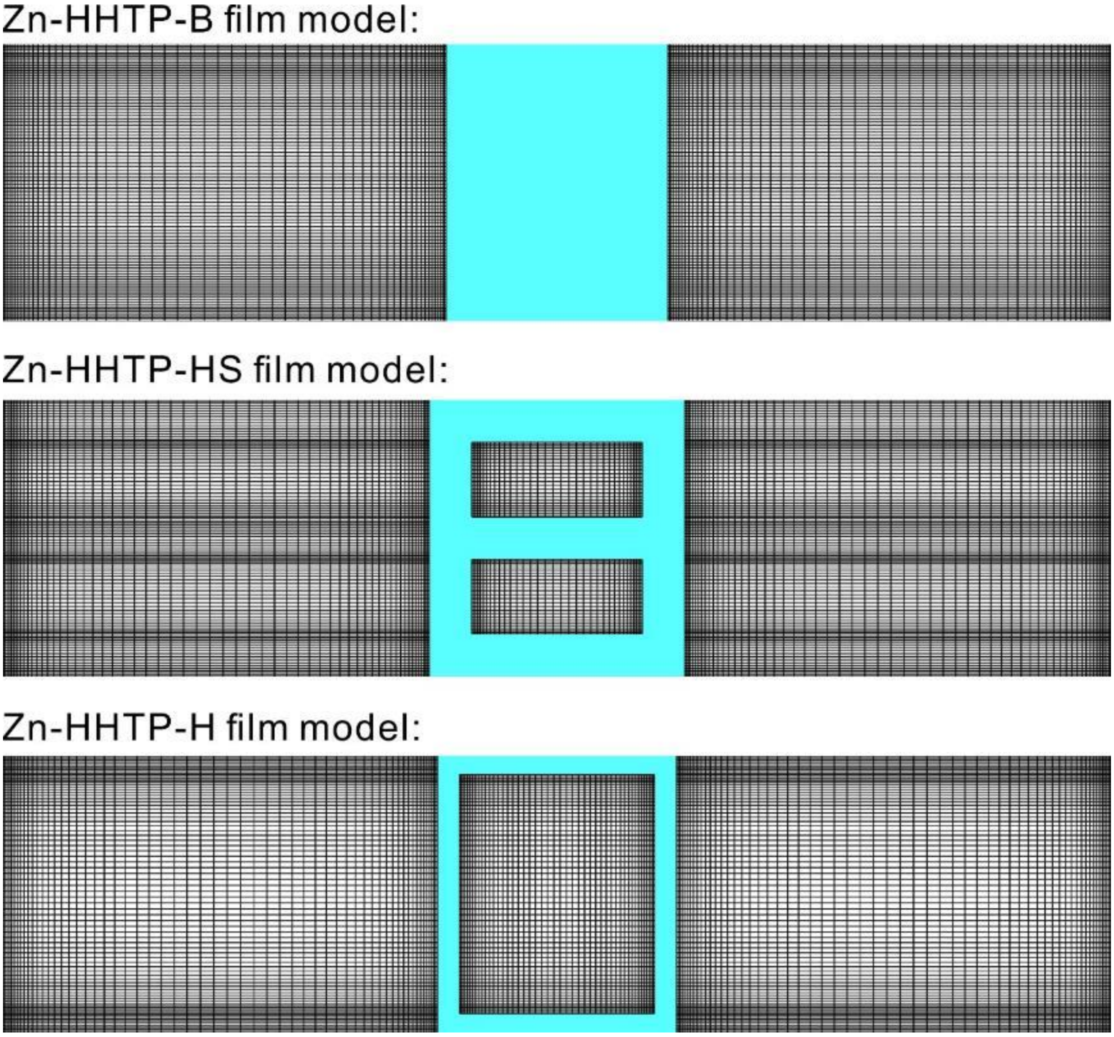

Supplementary Figure 30. Mesh distributions in the symmetry plane of a square channel with Zn-HHTP porous media. The mesh is refined at the Zn-HHTP film surfaces.

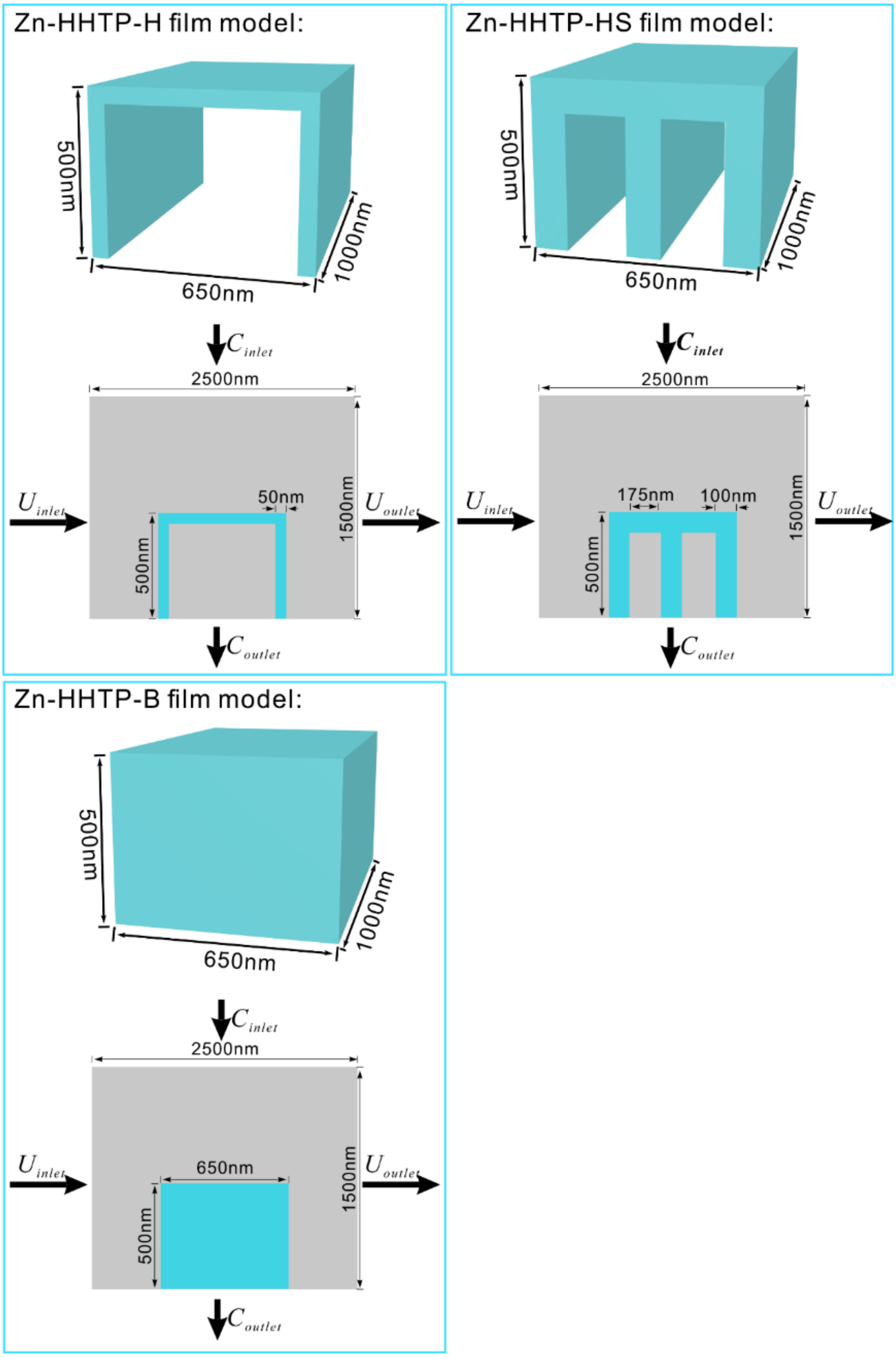

Supplementary Figure 31. Simulation models setup for the Zn-HHTP porous media. The left boundary of the channel is set as the inlet boundary while the right boundary as the exit boundary condition. For transport of reaction component, the top wall of channel is set as the concentration inlet boundary.

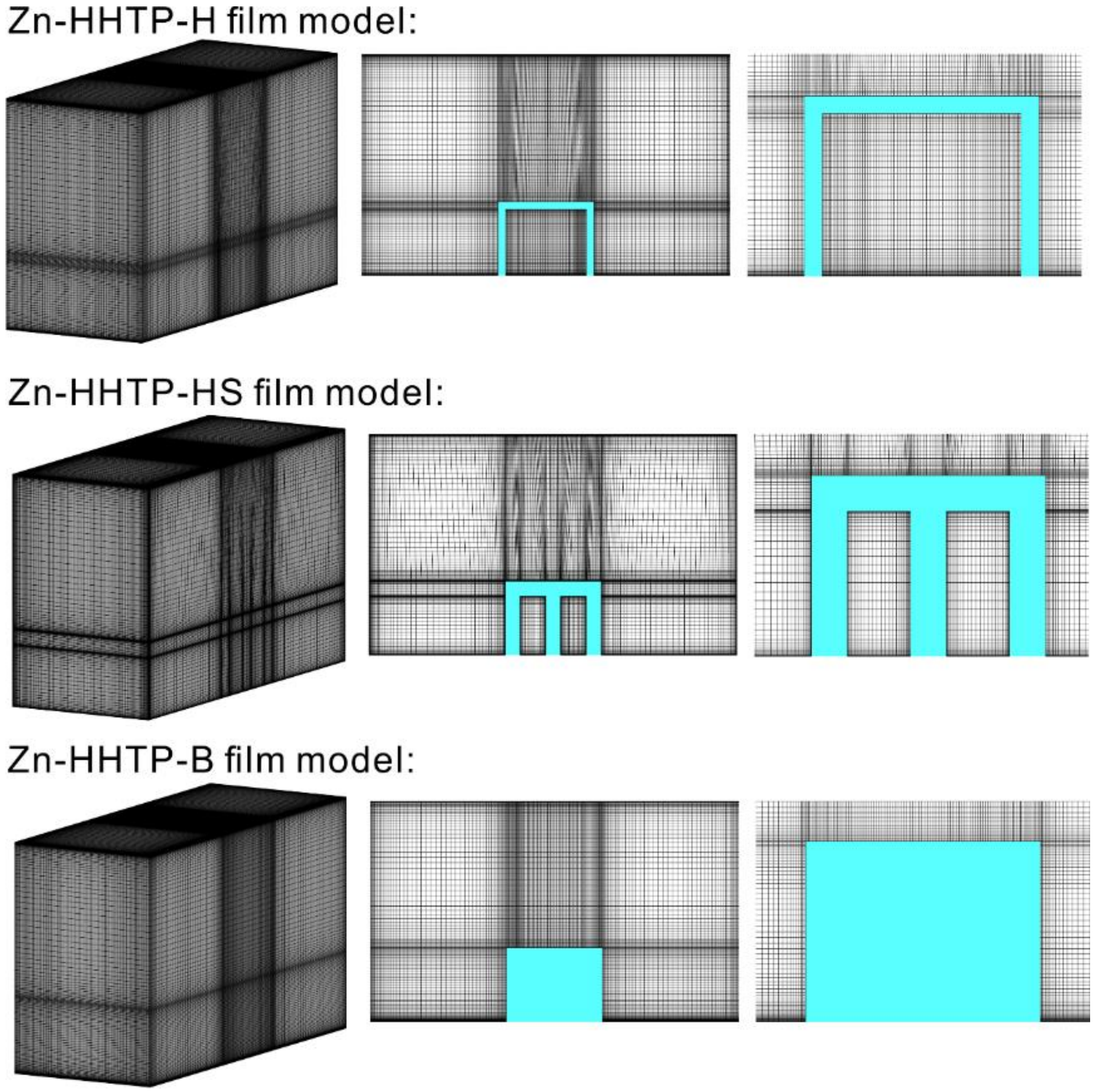

Supplementary Figure 32. 3D computational domain employing a hexahedral non-uniform structured mesh. The mesh is refined at the Zn-HHTP film surfaces.

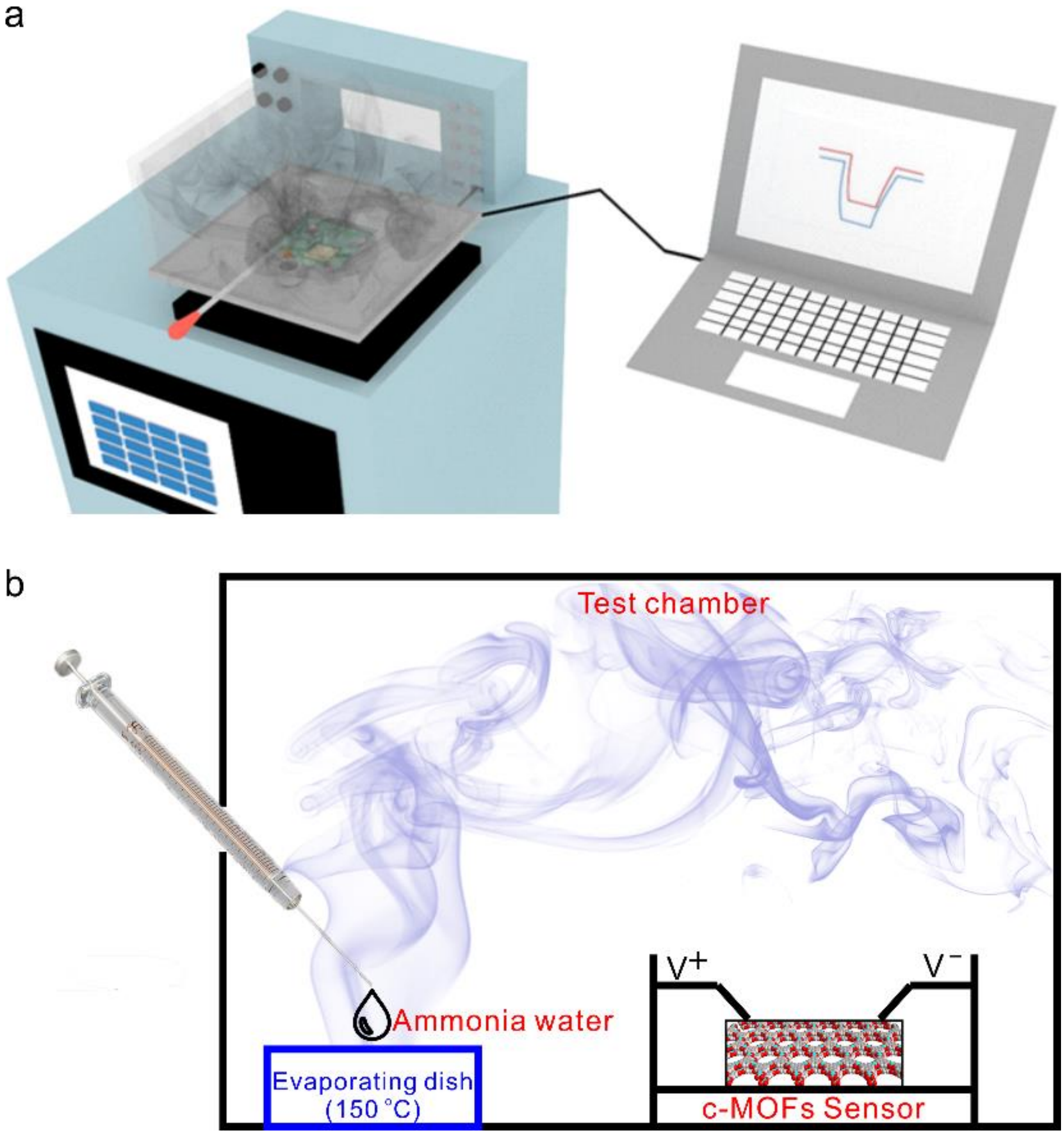

Supplementary Figure 33. (a) Diagram of the gas-sensing test device and (b) schematic diagram of airflow diffusion.

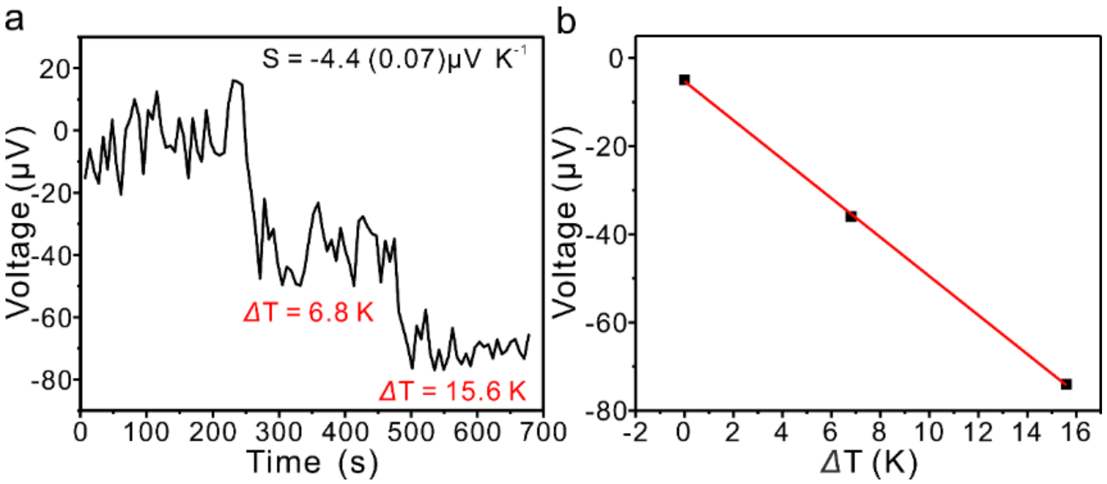

Supplementary Figure 34. (a) Temperature difference dependent thermal voltage and current in thermal voltage measurement for Zn-HHTP-H film. (b) The fitting line of temperature difference dependent thermal voltage of Zn-HHTP-H film.

As the Seebeck coefficient value is -4.4 ± 0.07 μV K^{-1}, revealing that the Zn-HHTP is N-type semiconductor[10].

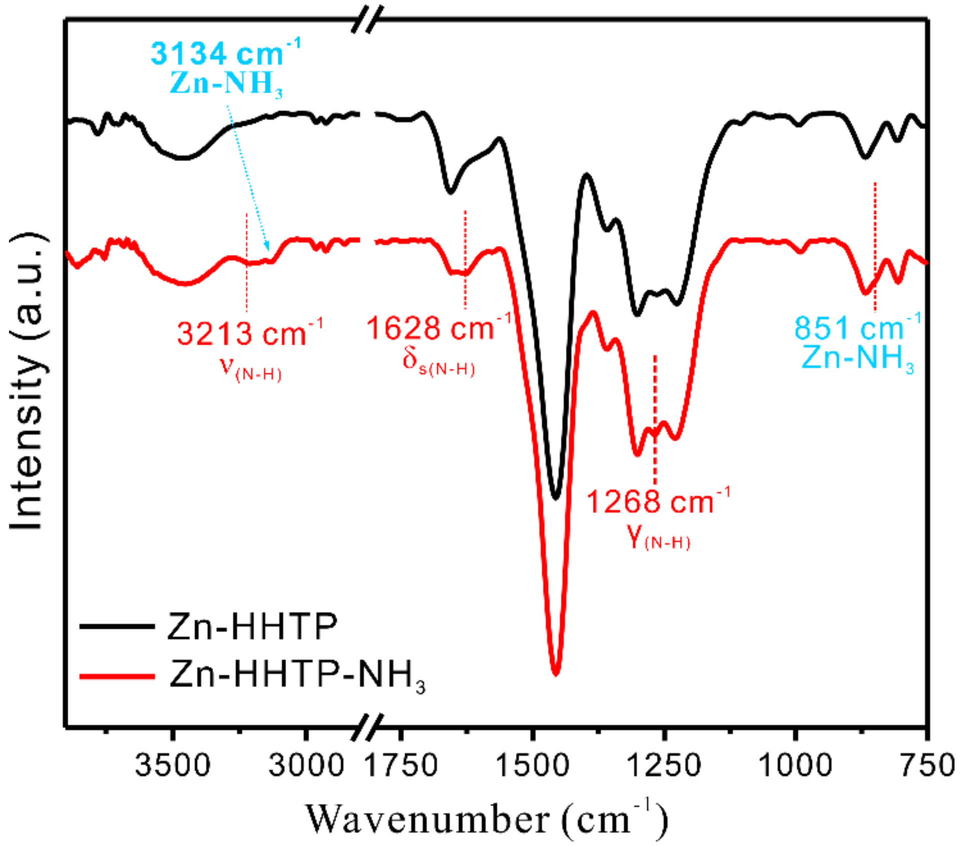

Supplementary Figure 35. FTIR spectra for the Zn-HHTP-H before and after the adsorption of NH_3.

The FTIR spectra show typical peaks for Zn-NH_3 as well as characteristic peaks of NH_2, suggesting strong interactions between NH_3 and the framework of $Zn_3(HHTP)_2$.

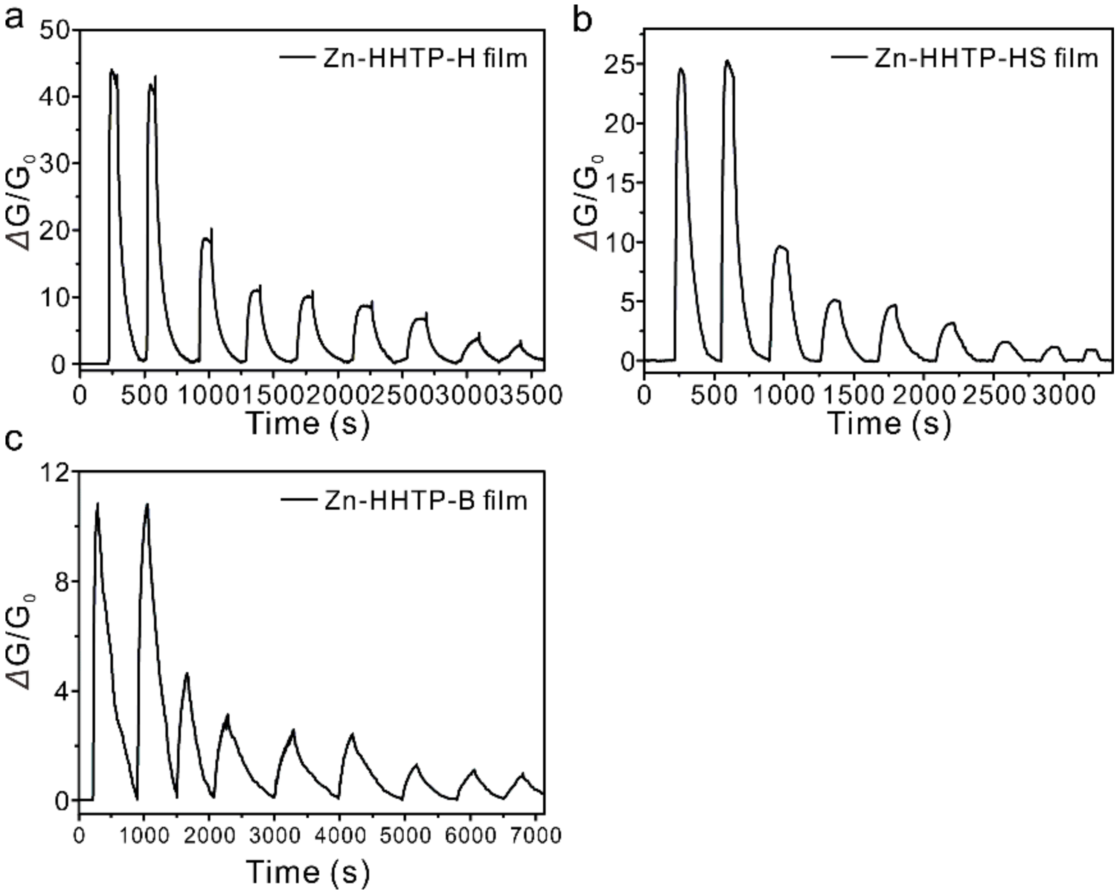

Supplementary Figure 36. (a, b and c) The response–recovery curve for Zn-HHTP films toward NH_3 with different concentrations. (From left to right, the concentrations are 50, 50, 20, 10, 8, 6, 4, 2 and 1 ppm, respectively.)

The Zn-HHTP-H sensor exhibited a response intensity of 43.8% toward 50 ppm ammonia while the response of Zn-HHTP-HS and Zn-HHTP-B films were only 24.8% and 10.8%, respectively. Such a high signal response of Zn-HHTP-H film should be attributed to two aspects. On the one hand, the Zn-HHTP-H film possessed higher exposed active sites than that of other Zn-HHTP films. Once exposed to ammonia with the same concentration, more gas molecules could be captured on Zn-HHTP-H film than other films. On the other hand, the signal transduction paths were significantly different on these Zn-HHTP films. For the Zn-HHTP-H film, the electron transfer path is mainly along the upper surface and this is just the position where the ammonia molecules adsorbed firstly. Therefore, the 20 nm thick upper surface with low electrical conductivity suffers a marked signal change upon exposed to ammonia, even at low gas concentration (Supplementary Table 5).

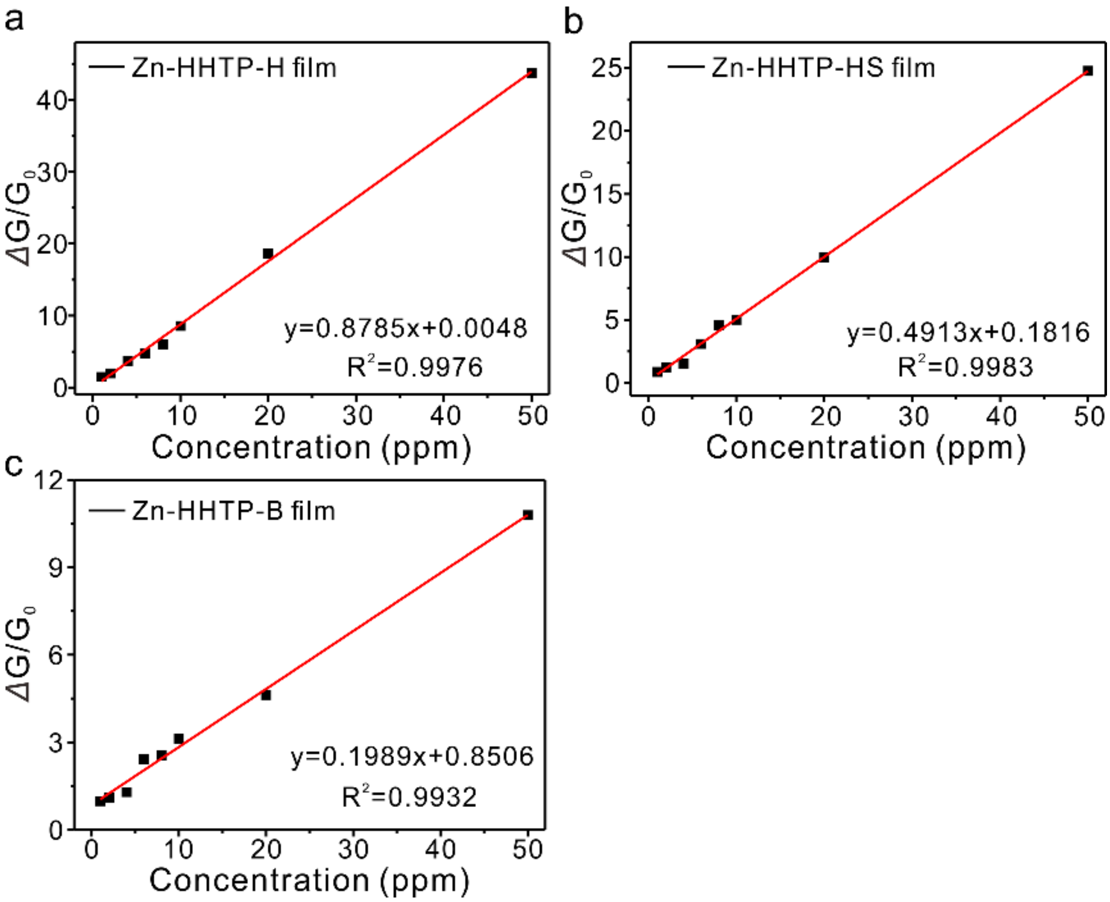

Supplementary Figure 37. (a, b and c) The linear fitting of the response-concentration plot for different for Zn-HHTP films.

Theoretical limit of detection (LOD) of Zn-HHTP-H sensor was calculated to be 39.9 ppb, which was also much lower than those of the other two Zn-HHTP sensors (108.2 ppb for Zn-HHTP-HS and 328.8 ppb for Zn-HHTP-B films).

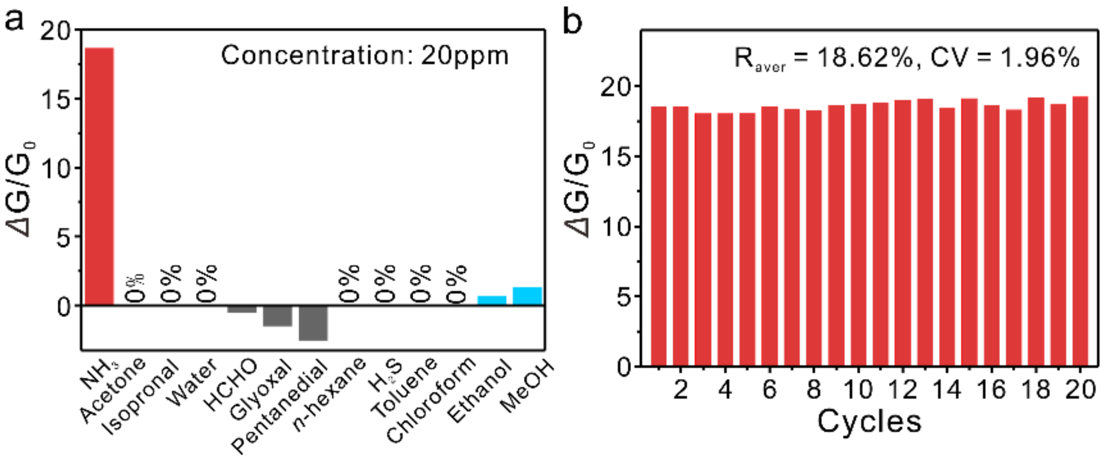

Supplementary Figure 38. (a) Selectivity of Zn-HHTP-H film-based NH_3 sensing platform towards 12 interference gases. (b) Repeated response for Zn-HHTP-H film-based NH_3 sensing platform towards 20 ppm NH_3.

Some common interferents, such as water, ethanol, toluene and acetone, exhibit no signal response on the Zn-HHTP-H film sensor. All the value of selectivity (S = Response (NH_3)/Response (gas)) of NH_3 toward other gases are more than 7.5, demonstrating Zn-HHTP film sensing platform is free from the interference of common gases in practical application. Besides, Zn-HHTP film exhibits good repeatability with low coefficient of variation (1.96 %) for repeated detection for 20 cycles (Figure 38b).

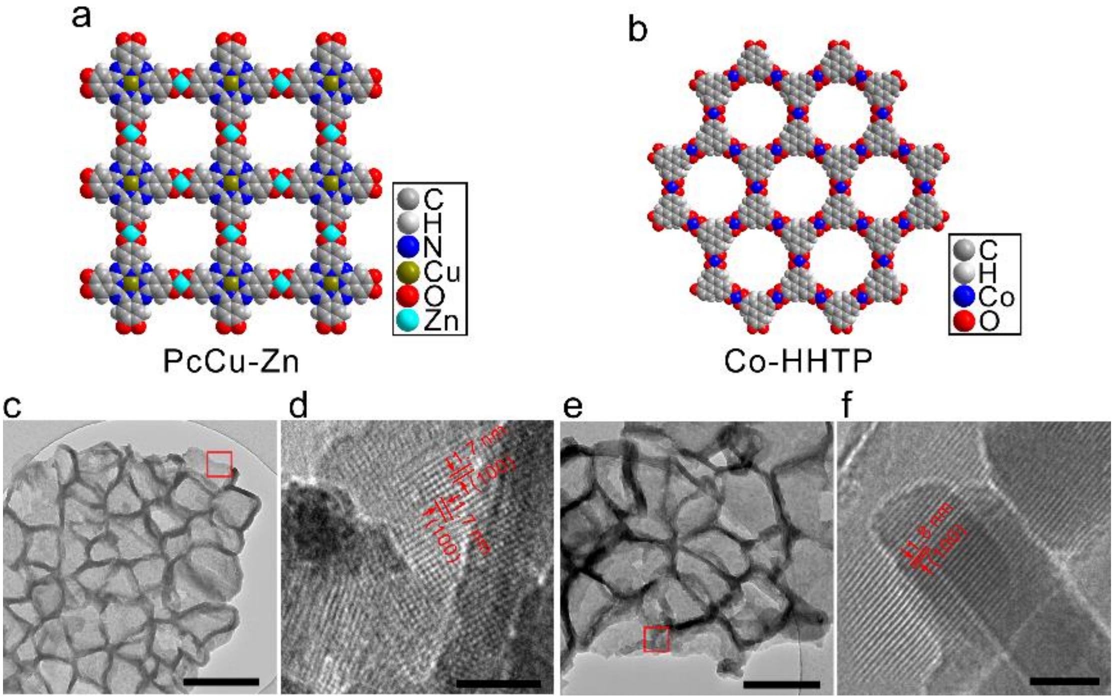

Supplementary Figure 39. (a) Schematic structure of PcCu-Zn MOFs. (b) Schematic structure of Co-HHTP MOFs films. (c) TEM image of the PcCu-Zn-H nanoparticles scraped off from the film. (d) High-resolution TEM image of the red square shown in (c). (e) TEM image of the Co-HHTP-H nanoparticles scraped off from the film. (f) High-resolution TEM image of the red square shown in (e). Scale bars represent 500 nm for (c and e), 20 nm for (d and f).

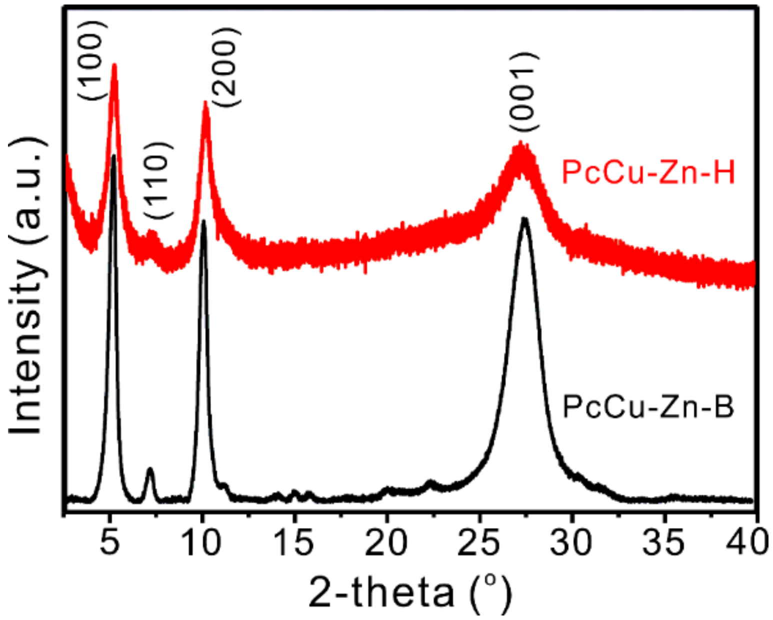

Supplementary Figure 40. Powder XRD patterns of the PcCu-Zn films.

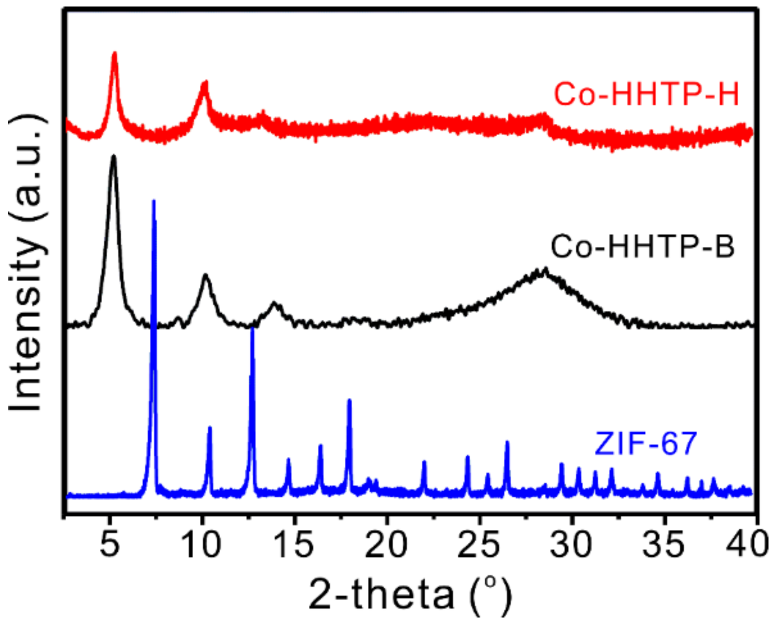

Supplementary Figure 41. Powder XRD patterns of the ZIF-67 and Co-HHTP films.

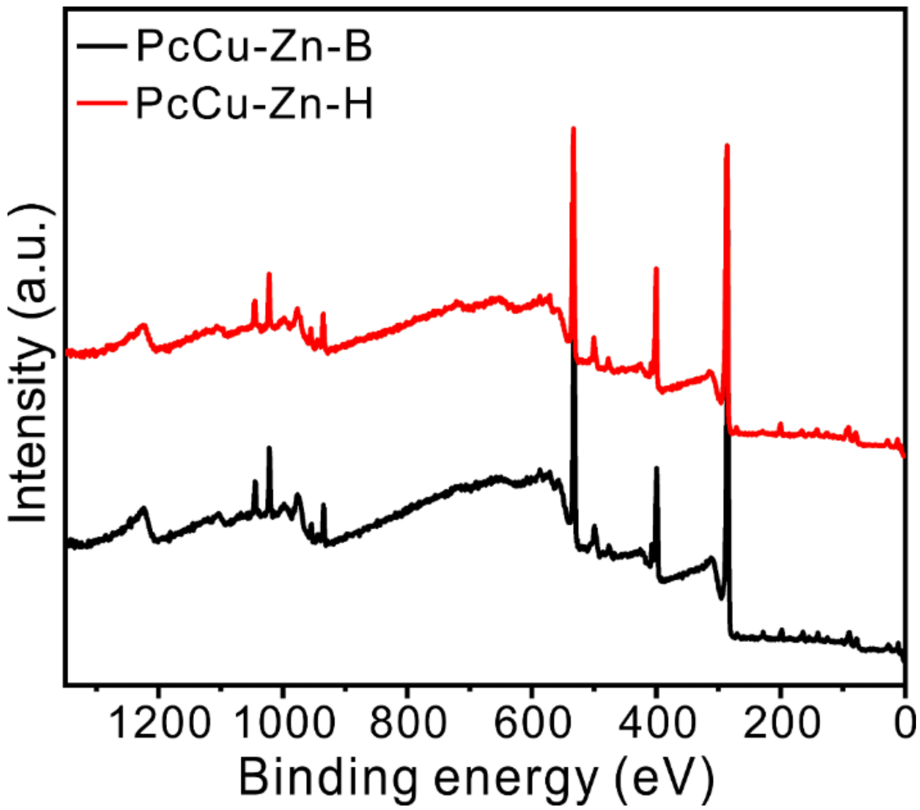

Supplementary Figure 42. XPS patterns of as-synthesized PcCu-Zn films.

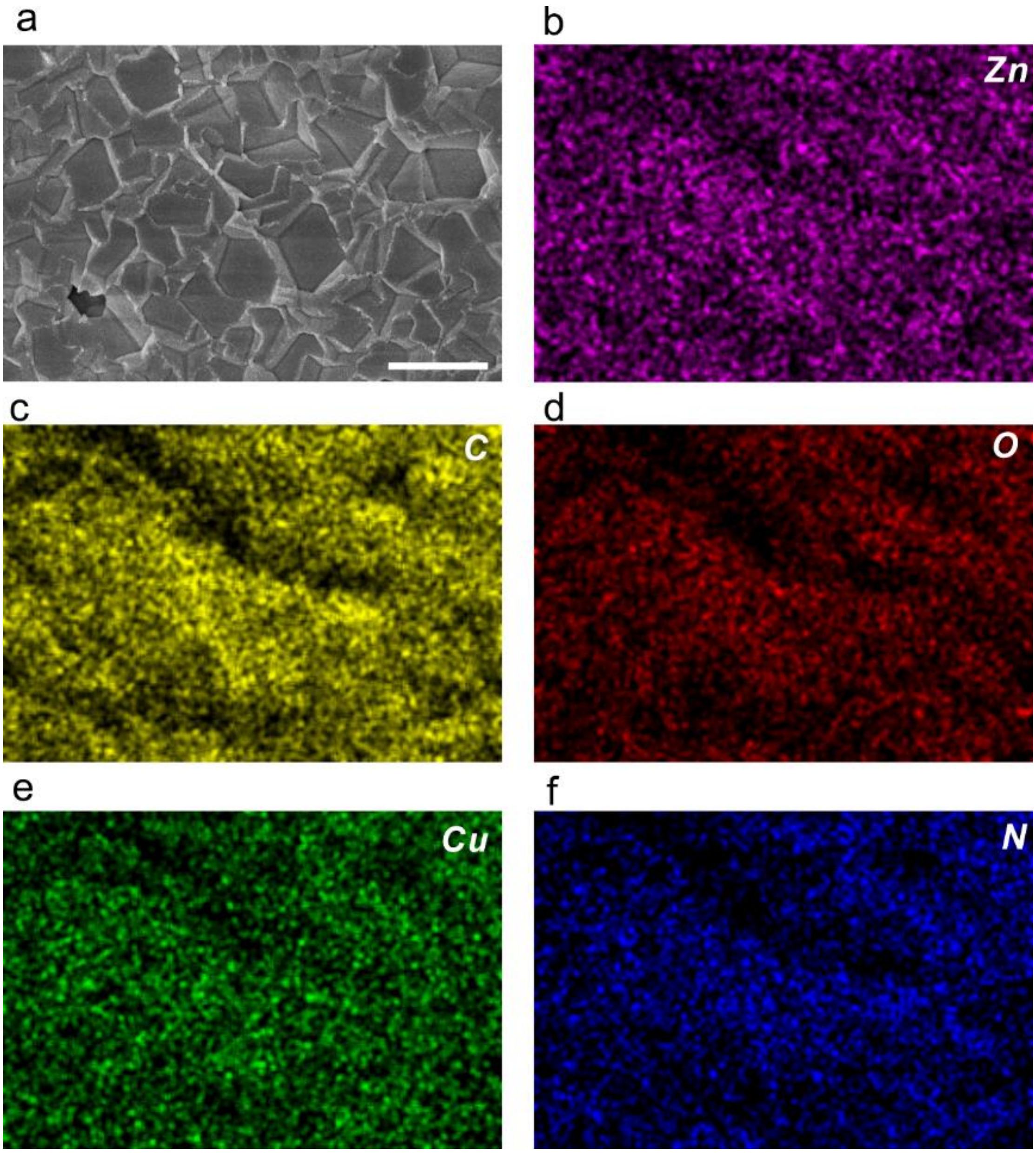

Supplementary Figure 43. (a-b) Surface SEM image of PcCu-Zn-H film. (c) The energy dispersive X-ray elemental mapping results for PcCu-Zn-H film. Scare bar represent 1 μm for (a).

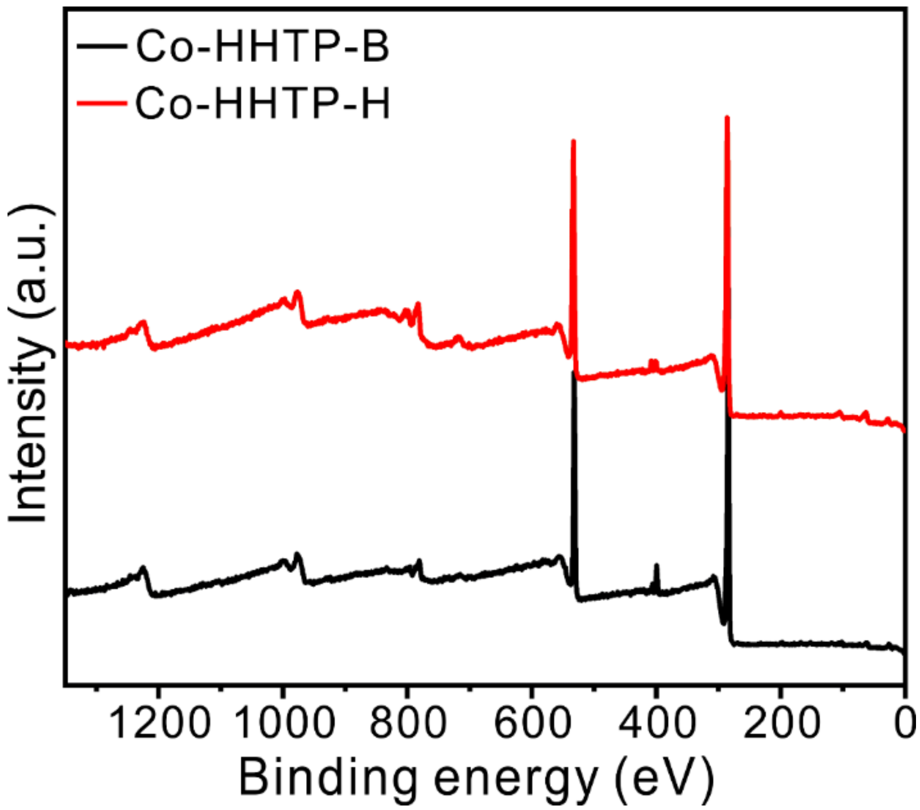

Supplementary Figure 44. XPS patterns of as-synthesized Co-HHTP films.

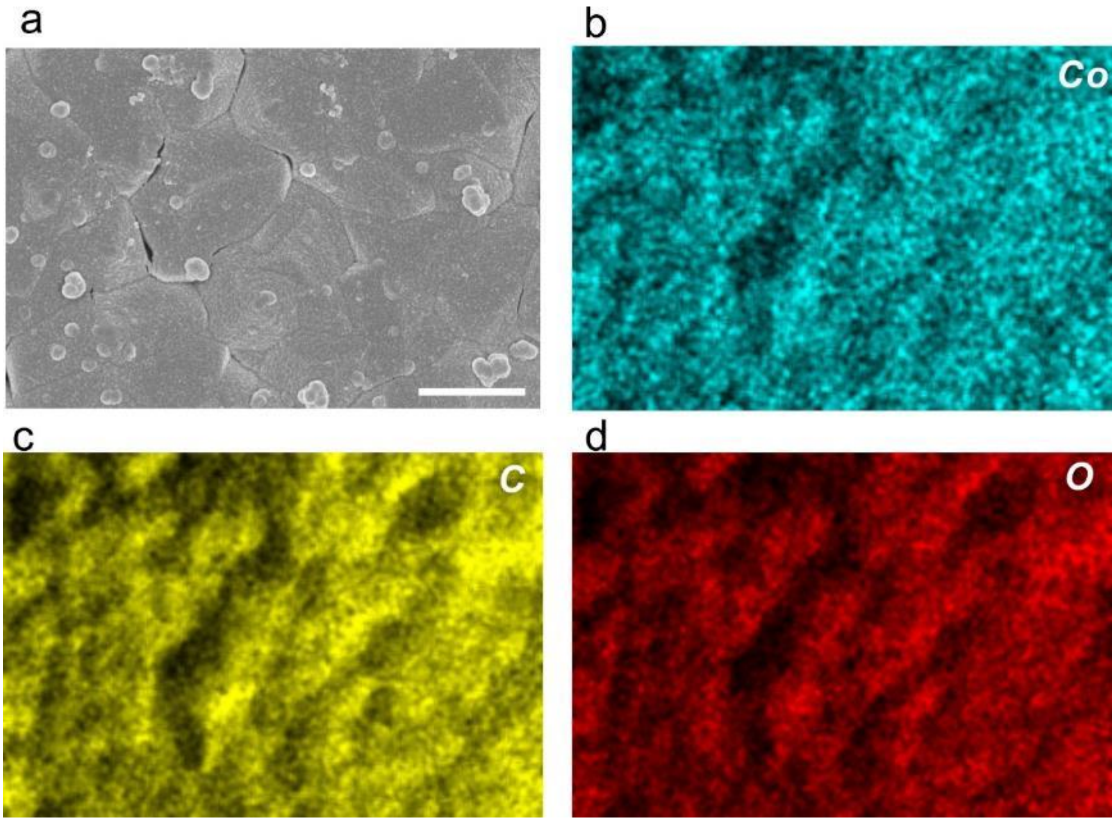

Supplementary Figure 45. (a-b) Surface SEM image of Co-HHTP-H film. (c) The energy dispersive X-ray elemental mapping results for Co-HHTP-H film. Scare bar represent 500 nm for (a).

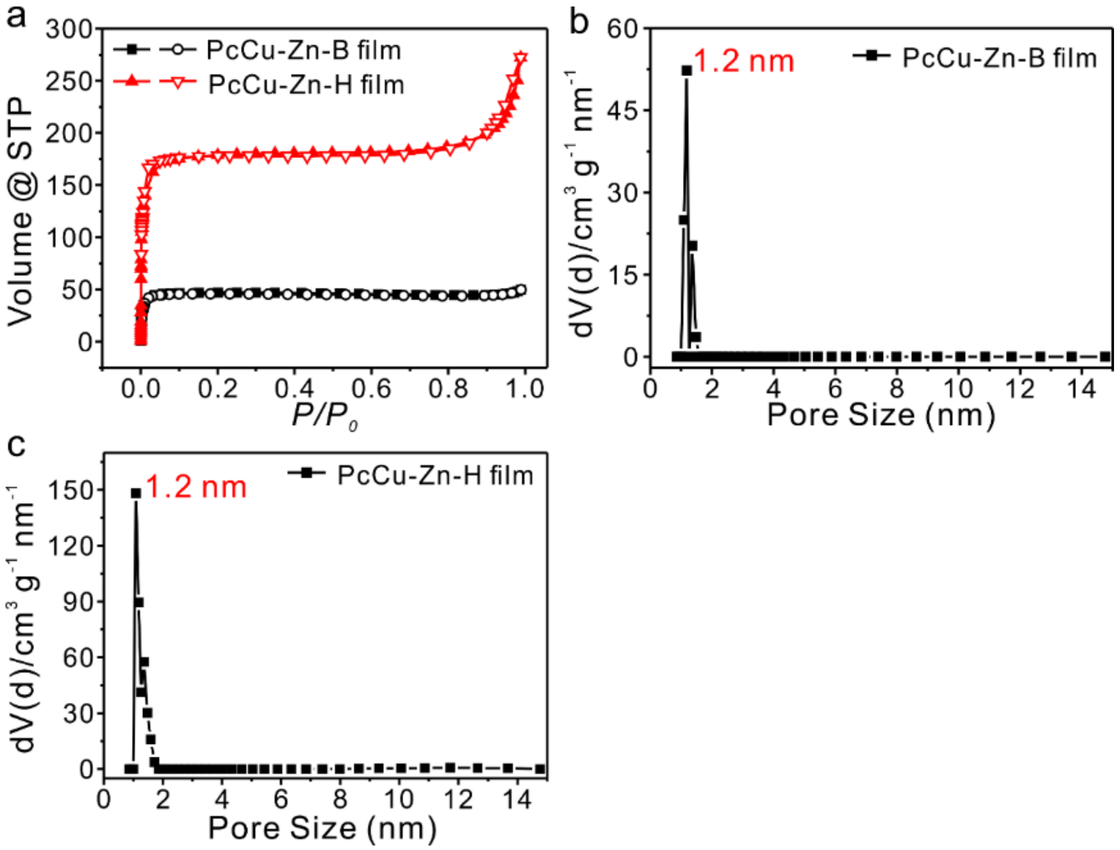

Supplementary Figure 46. (a) Nitrogen adsorption and desorption isotherms measured at 77 K of as-synthesized PcCu-Zn MOFs films. (b-d) The corresponding pore size distributions of the PcCu-Zn MOFs films.

The BET surface area of PcCu-Zn-H and PcCu-Zn-B films are 677.4 and 187.0 $m^2\ g^{-1}$, respectively.

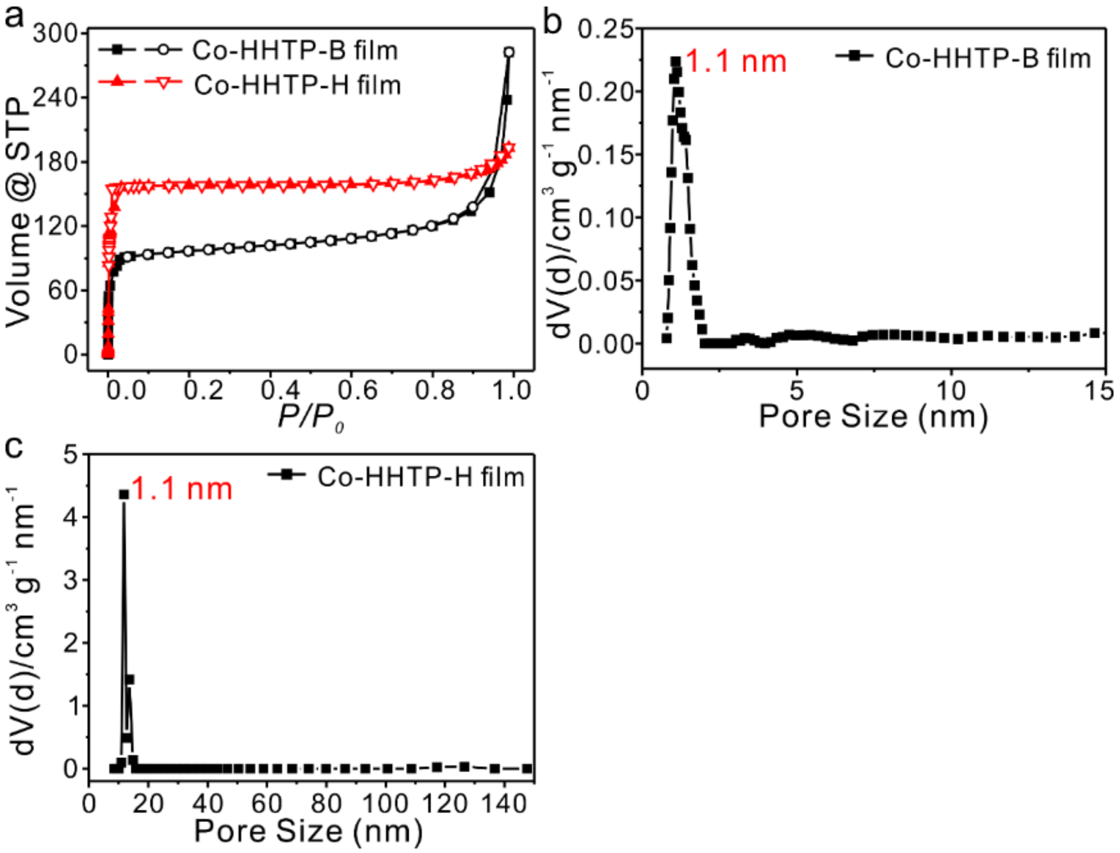

Supplementary Figure 47. (a) Nitrogen adsorption and desorption isotherms measured at 77 K of as-synthesized Co-HHTP MOFs films. (b-d) The corresponding pore size distributions of the Co-HHTP MOFs films.

The BET surface area of Co-HHTP-H and Co-HHTP-B films are 637.9 and 234.2 m^2 g^{-1}, respectively.

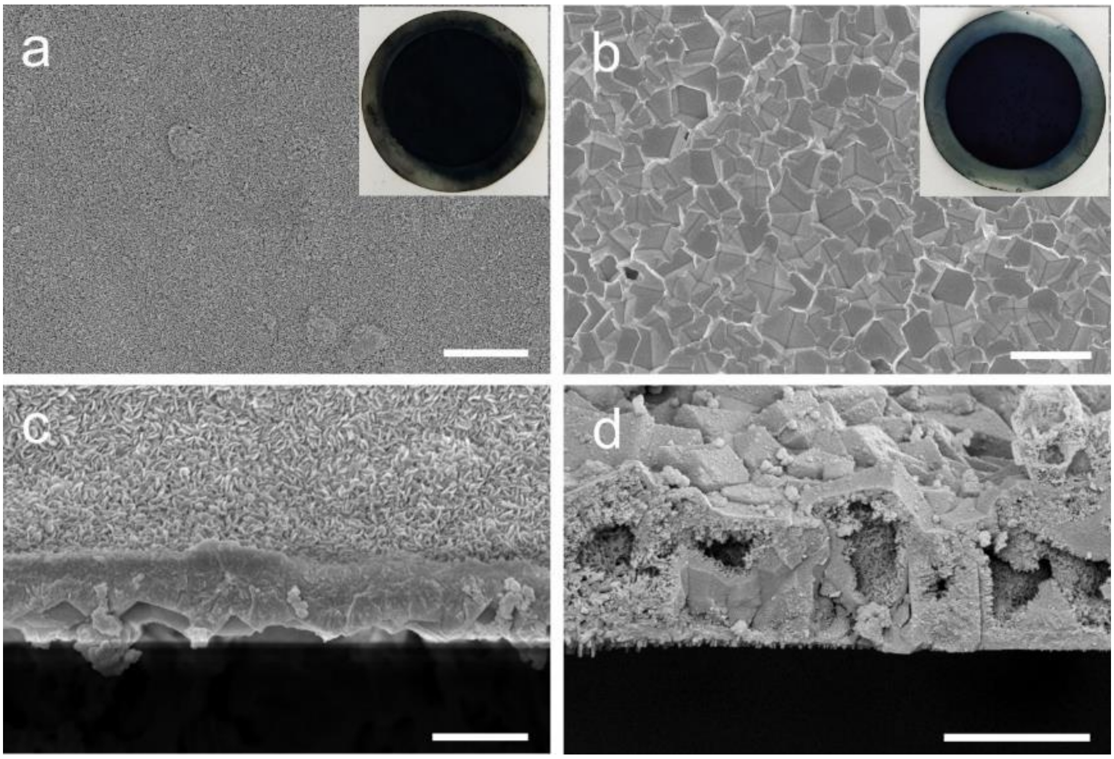

Supplementary Figure 48. (a, b) Surface SEM images of the PcCu-Zn-B and PcCu-Zn-H thin film on nylon 66 membrane, respectively. The insets are photographs of PcCu-Zn-B and PcCu-Zn-H coated nylon 66 membrane, respectively. (c-d) Cross-section SEM images of the PcCu-Zn-B and PcCu-Zn-H thin film on nylon 66 membrane, respectively. Scare bars were 1 μm for (a, b), 500 nm for (c, d).

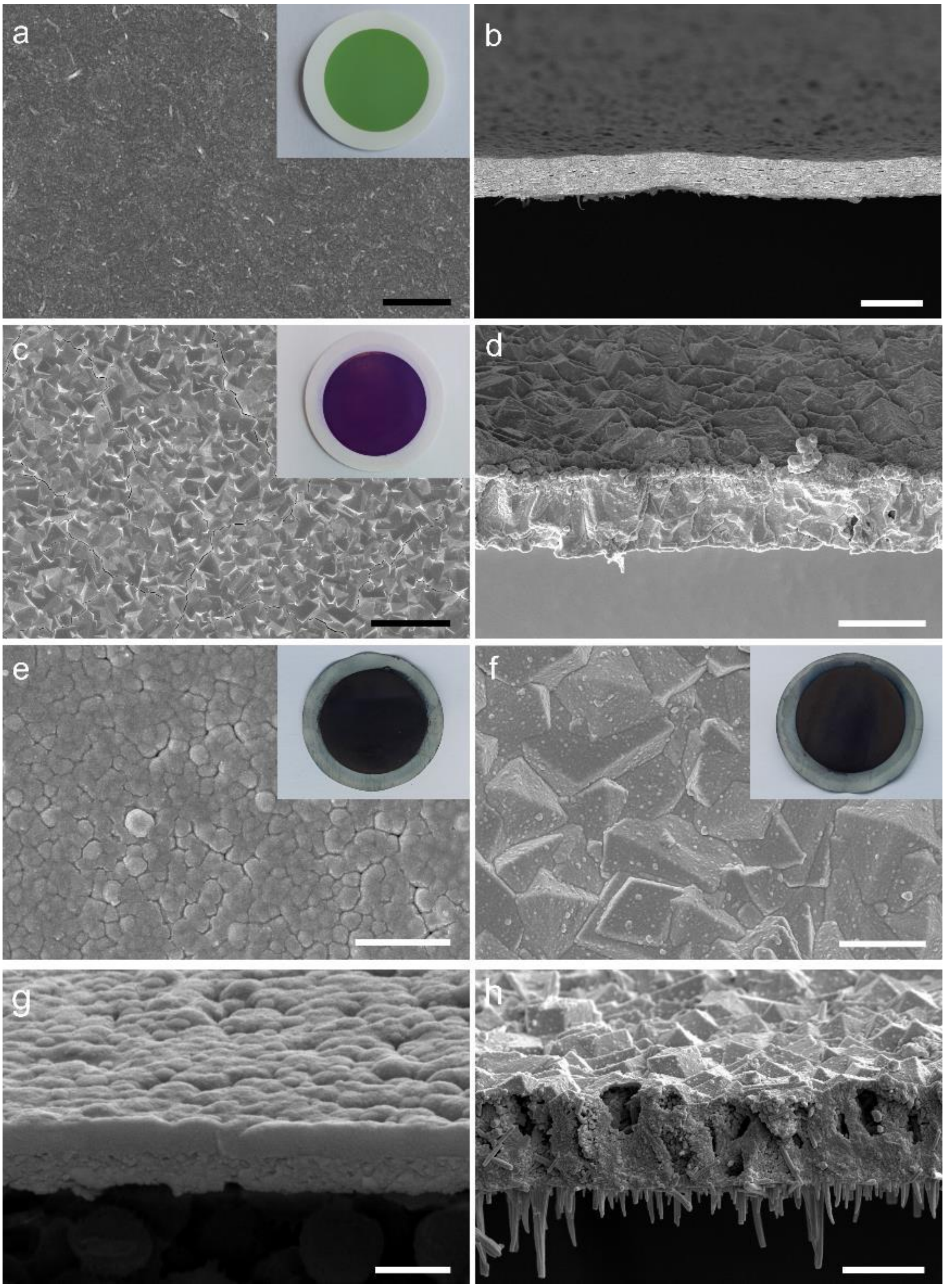

Supplementary Figure 49. (a-b) Surface and cross-section SEM images of the cobalt hydroxide nanostrands thin film on nylon 66 membrane. (c-d) Surface and cross-section SEM images of the ZIF-67 thin film on nylon 66 membrane. (e, g) Surface and cross-section SEM images of the Co-HHTP-B thin film on nylon 66 membrane. (f, h) Surface and cross-section SEM images of the Co-HHTP-H thin film on nylon 66 membrane. The insets in a, c, e and f are the photographs of cobalt hydroxide nanostrands, ZIF-67, Co-HHTP-B and Co-HHTP-H coated nylon 66 membrane, respectively. Scare bars were 1 μm for (a), 500 nm for (b), 2 μm for (c), 500 nm for (d), 1 μm for (e), 500 nm for (f), 500 nm for (g) and (h).

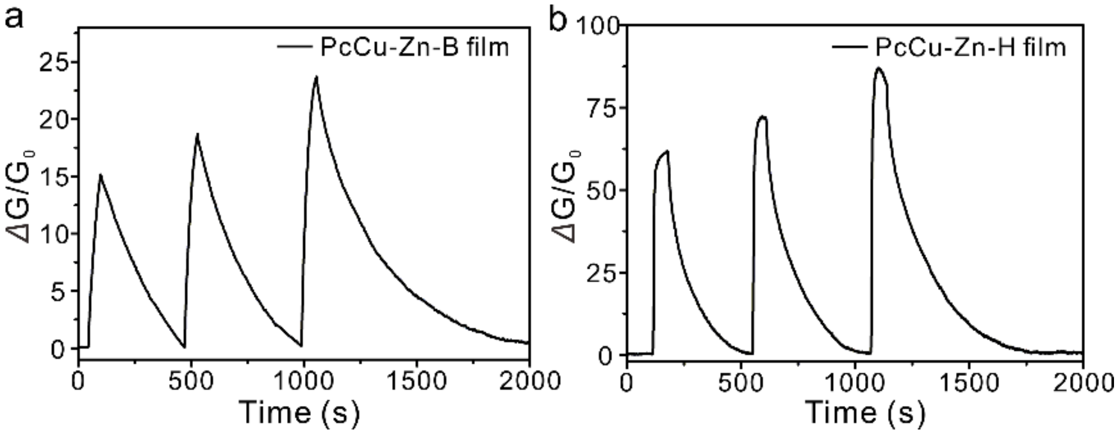

Supplementary Figure 50. (a and b) The response–recovery curve for PcCu-Zn films toward NH_3 with different concentrations. (From left to right, the concentrations are 100, 120, 150 ppm, respectively.)

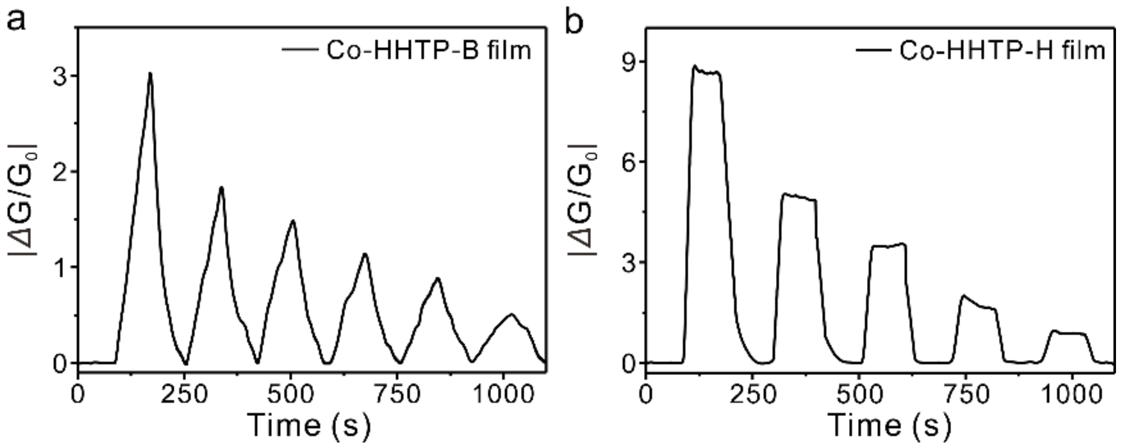

Supplementary Figure 51. (a and b) The response–recovery curve for Co-HHTP films toward NH_3 with different concentrations. (From left to right, the concentrations are 20, 15, 10, 5, 2 ppm, respectively)

Supplementary Table 1. Models and corresponding free energy used to calculate Gibbs free energy of formation of Zn-HHTP crystal from ZIF-8 precursor.

ZIF-8 (au)	HHTP (au)	Zn-HHTP (au)	Hmim (au)	ΔG* (kcal/mol)
-1125.705229	-1141.869382	-2349.216748	-265.158924	-256.3068828
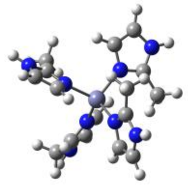	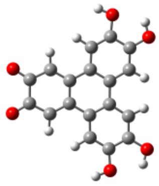	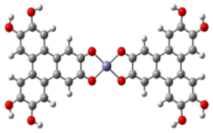	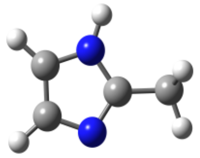	

*ΔG = [4*G(Hmim) + G(Zn-HHTP) - 2*G(HHTP) - G(ZIF-8)] *627.5095 kcal/mol.

Supplementary Table 2. The electrical conductivity of different *c*-MOFs plates was measured at room temperature. Conductivity of the MOF measured by four-point probe method.

Films	Conductivity (S/cm)
Zn-HHTP-H	1.94E-05
Zn-HHTP-HS	8.51E-05
Zn-HHTP-B	1.23E-04
PcCu-Zn-H	2.73E-06
PcCu-Zn-B	2.03E-04
Co-HHTP-H	6.21E-06
Co-HHTP-B	7.03E-04

Supplementary Table 3. Computational parameters for porous media model and Langmuir kinetic model.

	Porous media model		Langmuir kinetic model
	ε^a	Surface area^a(m^2 g^{-1})	k_a(m s^{-1}), k_d (s^{-1})
Zn-HHTP-B	0.2	165.3	0.001, 10^{-8}
Zn-HHTP-HS	0.3	479.4	0.001, 10^{-8}
Zn-HHTP-H	0.3	614.0	0.001, 10^{-8}

a: data from the BET gas adsorption measurement.

Supplementary Table 4. Calculation of modified permeability K_{hollow} from simulated results.

	β	$\frac{H^2 + 2K\alpha H}{1 + \alpha H}$	$\frac{2K\alpha - \alpha H^2}{1 + \alpha H}$	U_s	K_{hollow}	$K / K_{Zn\text{-}HHTP\text{-}B}$ (simulated)	$K / K_{Zn\text{-}HHTP\text{-}B}$ (Experimental)
Zn-HHTP-B	0	9.31×10^{-15}	-2.46×10^{-6}	4.87×10^{-6}	3.27×10^{-15}	1.00	1.00
Zn-HHTP-HS	0.43	1.36×10^{-14}	-1.28×10^{-6}	1.43×10^{-5}	6.01×10^{-15}	1.84	2.27
Zn-HHTP-H	0.76	7.06×10^{-14}	-1.69×10^{-6}	3.94×10^{-5}	2.88×10^{-14}	8.81	8.36

The values of K_{hollow} for Zn-HHTP-HS and Zn-HHTP-H films are calculated by fitting the relationship between the averaged velocity U and z obtained from the simulated results in the form of equation (6), and the related parameters are listed in Supplementary Table 4. Here, K_{bulk} is set to 2×10^{-15} m^2 in our porous medium model.

Supplementary Table 5. Comparison of room-temperature gas sensing performance toward NH_3 of various chemiresistive materials.

**Materials**	**Sensitivity (ppm^{-1})**	**Response time(s)**	**limit of detection (ppb)**	**Recovery time(min)**	**References**
Zn-HHTP-H film	0.81 (50 ppm)	9.1	39.9	0.99	**This work**
Zn-HHTP-HS film	0.495 (50 ppm)	41.9	108.2	1.36	**This work**
Zn-HHTP-B film	0.216 (50 ppm)	99.3	328.7	6.3	**This work**
PcCu-Zn-H film	0.618 (100 ppm)	9.8	-	3.64	**This work**
PcCu-Zn-B film	0.151 (100 ppm)	53.1	-	5.28	**This work**
Co-HHTP-H film	0.443 (20 ppm)	19.2	-	0.76	**This work**
Co-HHTP-B film	0.150 (20 ppm)	75.1	-	0.86	**This work**
Polyaniline	2.5 (5 ppm)	300	200	15	[11]
CuBHT	0.15 (100 ppm)	58	230	~119	[12]
COF-DC-8	0.975 (40 ppm)	90	57	1.7	[13]
NiPc-Ni MOF film	~0.181 (80 ppm)	90	310	>30	[7]
NiPc-Cu MOF film	~0.375 (80 ppm)	90	330	>30	[7]
PANInanofiber/ WS_2 nanosheet	0.405 (200 ppm)	260	50000	>30	[14]
HMP-TAPB-1	1.4 (50 ppm)	65	1000	13.2	[15]
V_2O_3 nanosheet	0.096 (25 ppm)	183	10000	0.15	[16]
$Cu_3(HHTP)_2$ thin film	1.29 (100 ppm)	81.6	500	1	[17]
BPB/R-GO	0.22 (25 ppm)	210	5000	9.11	[18]
$Cu_3(HHTP)_2$ nanorod film	0.07 (80 ppm)	~180	2500	~60	[19]
$Cu_3(HITP)_2$	~0.27 (10 ppm)	30	500	10	[20]

$CuTCNQF_4$	0.044 (99 ppm)	~300	25000	>6	[21]
N-doped CuO	0.01 (50 ppm)	~250	10000	>13	[22]
perylene diimide thin film	~0.34 (100 ppm)	28	560	N.A.	[23]
Pt activated SnO_2	0.31443 (1000 ppm)	75	50000	0.67	[24]
dialkyl tetrathiapentacene	10 (100 ppm)	31	10000	1.12	[25]
MoS_2 thin films	0.27 (30 ppm)	120	300	N.A.	[26]

References

1. Adamo, C. & Barone, V. Toward reliable density functional methods without adjustable parameters: The PBE0 model. *J. Chem. Phys.* **110**, 6158-6170 (1999).
2. Frisch, M. J.; Trucks, G. W.; Schlegel, H. B. *et al.* Gaussian 16 Rev. A.03. Wallingford, CT, (2016).
3. Dennington, R., Keith, T. A. & Millam, J. M. GaussView 6.0. 16. *Semichem Inc.: Shawnee Mission, KS, USA* (2016).
4. Mao, Y. *et al.* Foldable interpenetrated metal-organic frameworks/carbon nanotubes thin film for lithium–sulfur batteries. *Nat. Commun.* **8**, 1-8 (2017).
5. Ammu, S. *et al.* Flexible, all-organic chemiresistor for detecting chemically aggressive vapors. *J. Am. Chem. Soc.* **134**, 4553-4556 (2012).
6. Li, J. *et al.* Carbon nanotube sensors for gas and organic vapor detection. *Nano Lett.* **3**, 929-933 (2003).
7. Meng, Z., Aykanat, A. & Mirica, K. A. Welding metallophthalocyanines into bimetallic molecular meshes for ultrasensitive, low-power chemiresistive detection of gases. *J. Am. Chem. Soc.* **141**, 2046-2053 (2018).
8. Han, C., Zhang, C., Tyminska, N., Schmidt, J. R. & Sholl, D. S. Insights into the stability of zeolitic imidazolate frameworks in humid acidic environments from first-principles calculations. *J. Phys. Chem. C* **122**, 4339-4348 (2018).
9. Pang, S. H., Han, C., Sholl, D. S., Jones, C. W. & Lively, R. P. Facet-specific stability of ZIF-8 in the presence of acid gases dissolved in aqueous solutions. *Chem. Mater.* **28**, 6960-6967 (2016).
10. Gong, C. *et al.* Fabrication and thermoelectric properties of Ca-Co-O ceramics with negative Seebeck coefficient. *Results Phys.* **9**, 1233-1238 (2018).
11. Mkhize, N., Murugappan, K., Castell, M. R. & Bhaskaran, H. Electrohydrodynamic jet printed conducting polymer for enhanced chemiresistive gas sensors. *J. Mater. Chem. C* **9**, 4591-4596 (2021).
12. Chen, X. *et al.* Ultrafast in situ synthesis of large-area conductive metal–organic frameworks on substrates for flexible chemiresistive sensing. *ACS Appl. Mater. Interfaces* **12**, 57235-57244 (2020).
13. Meng, Z., Stolz, R. M. & Mirica, K. A. Two-dimensional chemiresistive covalent organic framework with high intrinsic conductivity. *J. Am. Chem. Soc.* **141**, 11929-11937 (2019).
14. Jha, R. K., Wan, M., Jacob, C. & Guha, P. K. Ammonia vapour sensing properties of in situ polymerized conducting PANI-nanofiber/WS_2 nanosheet composites. *New J. Chem.* **42**, 735-745 (2018).
15. Sharma, N. *et al.* Heptazine based organic framework as a chemiresistive sensor for ammonia detection at room temperature. *J. Mater. Chem. A* **6**, 18389-18395 (2018).
16. Mounasamy, V. *et al.* Template-free synthesis of vanadium sesquioxide (V_2O_3) nanosheets and their room-temperature sensing performance. *J. Mater. Chem. A* **6**, 6402-6413 (2018).
17. Yao, M. S. *et al.* Layer-by-layer assembled conductive metal–organic framework nanofilms for room-temperature chemiresistive sensing. *Angew. Chem., Int. Ed.* **129**, 16737-16741 (2017).
18. Duy, L. T. *et al.* Flexible transparent reduced graphene oxide sensor coupled with organic dye molecules for rapid dual-mode ammonia gas detection. *Adv. Funct. Mater.* **26**, 4329-4338 (2016).
19. Smith, M. K., Jensen, K. E., Pivak, P. A. & Mirica, K. A. Direct self-assembly of conductive nanorods of metal–organic frameworks into chemiresistive devices on shrinkable polymer films. *Chem. Mater.* **28**, 5264-5268 (2016).
20. Campbell, M. G., Sheberla, D., Liu, S. F., Swager, T. M. & Dincă, M. Cu_3 (hexaiminotriphenylene)$_2$: an electrically conductive 2D metal–organic framework for chemiresistive sensing. *Angew. Chem., Int. Ed.* **54**, 4349-4352 (2015).
21. Hoshyargar, F., Shafiei, M., Piloto, C., Motta, N. & O'Mullane, A. P. Investigation of the room temperature gas sensing properties of metal–organic charge transfer complex $CuTCNQF_4$. *J. Mater. Chem. C* **4**, 11173-11179 (2016).
22. Shafiei, M. *et al.* Conversion of n-type CuTCNQ into p-type nitrogen-doped CuO and the implication for room-temperature gas sensing. *J. Phys. Chem. C* **119**, 22208-22216 (2015).
23. Kalita, A., Hussain, S., Malik, A. H., Subbarao, N. V. & Iyer, P. K. Vapor phase sensing of ammonia at the sub-ppm level using a perylene diimide thin film device. *J. Mater. Chem. C* **3**,

10767-10774 (2015).
24. Liu, X. *et al.* Nanoparticle cluster gas sensor: Pt activated SnO_2 nanoparticles for NH_3 detection with ultrahigh sensitivity. *Nanoscale* **7**, 14872-14880 (2015).
25. Li, L. *et al.* High performance field-effect ammonia sensors based on a structured ultrathin organic semiconductor film. *Adv. Mater.* **25**, 3419-3425 (2013).
26. Chabukswar, V., Pethkar, S. & Athawale, A. A. Acrylic acid doped polyaniline as an ammonia sensor. *Sens. Actuators B Chem.* **77**, 657-663 (2001).
