# Supplementary material for: Hierarchical conductive metal-organic framework films enabling efficient interfacial mass transfer
Source: Nat Commun. 2023 Jun 29;14:3850. doi: 10.1038/s41467-023-39630-y (PMC10310809; doi:10.1038/s41467-023-39630-y)
Supplement: Supplementary file 4 — Description of Additional Supplementary Files [file 41467_2023_39630_MOESM4_ESM.pdf]

Description of Additional Supplementary Files

Supplementary Movie 1
Description: The time evolutions of NH3 concentration distributions on the surface of Zn-HHTP-B film model (from 0 to 0.3 s)

Supplementary Movie 2
Description: The time evolutions of NH3 concentration distributions on the surface of Zn-HHTP-H film model (from 0 to 0.3 s)

Supplementary Movie 3
Description: . The time evolutions of NH3 concentration distributions on the surface of Zn-HHTP-HS film model (from 0 to 0.3 s).
